# Divergent Molecular and Cellular Responses to Low and High-Dose Ionizing Radiation

**DOI:** 10.3390/cells11233794

**Published:** 2022-11-27

**Authors:** Bharath Sampadi, Sylvia Vermeulen, Branislav Mišovic, Jan J. Boei, Tanveer S. Batth, Jer-Gung Chang, Michelle T. Paulsen, Brian Magnuson, Joost Schimmel, Hanneke Kool, Cyriel S. Olie, Bart Everts, Alfred C. O. Vertegaal, Jesper V. Olsen, Mats Ljungman, Penny A. Jeggo, Leon H. F. Mullenders, Harry Vrieling

**Affiliations:** 1 Department of Human Genetics, Leiden University Medical Center, 2333ZC Leiden, The Netherlands; 2 Proteomics Program, Novo Nordisk Foundation Center for Protein Research, Faculty of Health and Medical Science, University of Copenhagen, Blegdamsvej 3B, 2200 Copenhagen, Denmark; 3 Department of Cell and Chemical Biology, Leiden University Medical Center, 2333ZC Leiden, The Netherlands; 4 Department of Radiation Oncology, Rogel Cancer Center and Center for RNA Biomedicine, University of Michigan, Ann Arbor, MI 48109, USA; 5 Department of Parasitology, Leiden University Medical Center, 2333ZA Leiden, The Netherlands; 6 Genome Damage and Stability Centre, School of Life Sciences, University of Sussex, Brighton BN1 9RQ, UK; 7 Department of Genetics, Research Institute of Environmental Medicine (RIeM), Nagoya University, Nagoya 464-8601, Japan

**Keywords:** ionizing radiation, low dose, linear no-threshold, signal transduction, cell signaling, DNA damage response, phosphoproteomics, mitochondria, reactive oxygen species, antioxidant response

## Abstract

Cancer risk after ionizing radiation (IR) is assumed to be linear with the dose; however, for low doses, definite evidence is lacking. Here, using temporal multi-omic systems analyses after a low (LD; 0.1 Gy) or a high (HD; 1 Gy) dose of X-rays, we show that, although the DNA damage response (DDR) displayed dose proportionality, many other molecular and cellular responses did not. Phosphoproteomics uncovered a novel mode of phospho-signaling via S12-PPP1R7, and large-scale dephosphorylation events that regulate mitotic exit control in undamaged cells and the G2/M checkpoint upon IR in a dose-dependent manner. The phosphoproteomics of irradiated DNA double-strand breaks (DSBs) repair-deficient cells unveiled extended phospho-signaling duration in either a dose-dependent (DDR signaling) or independent (mTOR-ERK-MAPK signaling) manner without affecting signal magnitude. Nascent transcriptomics revealed the transcriptional activation of genes involved in NRF2-regulated antioxidant defense, redox-sensitive ERK-MAPK signaling, glycolysis and mitochondrial function after LD, suggesting a prominent role for reactive oxygen species (ROS) in molecular and cellular responses to LD exposure, whereas DDR genes were prominently activated after HD. However, how and to what extent the observed dose-dependent differences in molecular and cellular responses may impact cancer development remain unclear, as the induction of chromosomal damage was found to be dose-proportional (10–200 mGy).

## 1. Introduction

Epidemiological studies provide strong evidence for the increased risk of developing solid cancers or leukemia from high doses (HD) of ionizing radiation (IR) [1]. Cancer risk after exposure to low doses (LD) of IR (<0.1 Gy) is assumed to be linear with the dosage, without a threshold, but definite evidence is lacking [2]. This outstanding issue is of societal importance because of growing concern over increased cancer risk for people from occupational and medical exposure to LD [3,4,5,6]. A notable example of adverse health effects is the very high proportion (>90%) of therapy-related (including radiotherapy) adverse health effects in survivors of pediatric cancers, including a broad spectrum of second primary tumors by the age of 45 years [7,8]. The uncertainty and controversy regarding LD health risks are primarily due to an insufficient mechanistic understanding of the molecular effects of LD exposure on cells, tissues, and organisms [9,10]. Stem and early progenitor cells are considered cells of origin for radiation-induced carcinogenesis, owing to their replicative potential and long lifespan in the organism [11]. Increasing evidence suggests that stem cells display distinct responses to low versus high-dose IR, including activation thresholds for proliferation and differentiation [12], the dose-dependent integration of extrachromosomal DNA after LD [13], low-dose-specific hyper-radiosensitivity [14], persistent oxidative stress and decreased self-renewal [15] and the selective stimulation of proliferation based on their p53 mutation status [16]. The survival/persistence of normal tissue stem cells with residual DNA damage may increase the risk of second primary tumors.

IR exposure in cells induces various types of DNA damage (lesions) either directly through energy deposition in the DNA or indirectly through reactive oxygen species (ROS) generated by the radiolysis of water molecules in cells [17]. Although DNA damage induction is linear with dose [18], ROS induction is non-linear and is enhanced specifically after LD exposure [12,15,16,18,19,20,21]. The crucial role of DNA damage in radiation carcinogenesis has been well established [22]. DNA double-strand breaks (DSBs) and clustered damage, comprising any induced lesion type in proximity to other induced lesions, are considered the most relevant forms of DNA damage for IR-induced cancers [23]. Upon DNA damage infliction, cells activate the DNA damage response (DDR), an extensive network of intracellular responses [24,25]. The DDR is initiated upon DNA damage detection by sensor proteins, which subsequently activate signaling cascades that guide downstream cellular processes, including transcription and replication, DNA repair, cell cycle arrest, differentiation and apoptosis. Protein phosphorylation plays a crucial role in sculpting DNA-damage-induced signaling cascades with protein kinases and phosphatases (de)phosphorylating a plethora of substrates. ATM, ATR and DNA-PKcs kinases (phosphoinositide 3-kinase (PI3K)-related kinases family members) play a pivotal role in DDR activation in mammals [26].

Dose-dependent differences in phosphorylation signaling are likely to critically modulate the magnitude and complexity of cellular responses after LD and HD. Phosphoproteome responses have been established for various DSB-inducing agents, such as HD IR [27,28,29], the topoisomerase inhibitor etoposide [30] and the radiomimetic chemical NCS [31]. In response to these treatments, activation of the ATM kinase is a major event leading to altered phosphorylation states in numerous substrates [27,32]. Furthermore, sustained ATM activity is required to maintain many ATM-dependent phosphorylations [31], implicating the high activity of counteracting phosphatases. Additionally, studies [28,31] have reported a substantial number of ATM-independent phosphorylation events, highlighting the involvement of several other kinases, such as ATR, DNA-PKcs and CSNK1A1/CSNK2A1 in DNA damage signaling. Bioinformatic analyses of HD-activated phosphoproteomes identified various affected cellular processes, including transcription and RNA processing, chromatin remodeling, DNA repair, cell cycle checkpoints and apoptosis [28,29,31]. These studies, however, represented either a snapshot of the phosphoproteomic changes at a single time point or were restricted to (S/T)Q motif-containing phosphosites or phosphosites in nuclear proteins. At the transcriptional level, ATM-activated Tp53 coordinates the regulation of several genes in the Tp53 signaling pathway [33,34,35,36,37,38]. It is, however, unclear to what extent low-dose irradiation affects global phosphorylation signaling cascades and de novo transcription, as these have scarcely been investigated [39,40].

In this study, we examined the IR dose–response relationship by performing an integrated in-depth systems analysis with a high temporal resolution to decipher molecular mechanisms underlying the cellular responses to low (0.1 Gy) (LD, 2–4 DSBs per cell) and high (1 Gy) (HD, 20–40 DSBs per cell) doses of X-rays [41] in mouse embryonic stem cells (mESCs). We explored the impact of IR dosage on the dynamic properties (duration, amplitude and kinetics) of phosphorylation signals and investigated how these signaling events were affected when DSB repair was impaired by employing DSB repair-deficient (DRD) cell lines. 

## 2. Materials and Methods

### 2.1. Cell Culture, Protein Labelling and Irradiation

The following mouse embryonic stem cell lines (mESC) (Cat# ATCC^®^ SCRC-1010™; LGC Standards GmbH, Wesel, Germany) were used: IB10 (E14.IB10) wild-type (wt), IB10 Lig4^−/−^ and IB10 Xrcc5^−/−^ [42,43]. Cell lines were cultured, passaged and triple-SILAC labelled according to our previously published protocol [44] with minor modifications, as follows. Cells were grown on top of a monolayer of lethally irradiated mouse embryonic fibroblasts for four passages and were subsequently passaged without feeders for a total of seven passages to complete SILAC labelling. The medium was washed from the cells with PBS (Cat# 14190086, Life Technologies, Carlsbad, CA, USA). Single-cell suspensions were produced using Trypsin-EDTA (0.05%) (Cat#25300-054, Life Technologies, Carlsbad, CA, USA). Cell lines were frequently verified to be mycoplasma-free. DNA damage was inflicted by exposing cells to 0.1 Gy of X-rays (dose-rate 0.1 Gy/min) for LD and to 1 Gy (dose-rate 1 Gy/min) for HD using the YXlon X-ray generator (YXLON International X-Ray GmbH—Hamburg, Germany, 200 KV). The culture dishes were placed on top of the inner chamber at the designated location during each irradiation event for both low- and high-dose exposures to prevent variations in the source–sample distance. We altered only the amount of current delivered to the X-ray generator to achieve either a low or a high dose while keeping all other parameters constant, including the source–sample distance and the time of exposure to IR.

### 2.2. Phosphoproteomics and Mass Spectrometry Measurements

Samples were prepared according to our previously published protocol [45]. Briefly, proteins in cell lysates were digested into peptides using trypsin/lys-c mix (Cat# V5072, Promega, Leiden, The Netherlands) in 500 μL of 10% trifluoroethanol (TFE; Cat# 05841-50 mL, Sigma, Amsterdam, The Netherlands) buffer. Subsequently, the digested peptides were diluted 3.2-fold to reach a final volume of 1.6 mL with final concentrations of 50% acetonitrile (can) and 6% trifluoroacetic acid (TFA) buffer with potassium salts (0.3 M KCl and 0.005 M KH_2_PO_4_), were enriched for phosphopeptides using TiO_2_ beads (Cat# 5010-21315, GL Sciences, Eindhoven, The Netherlands), were washed six times with 60% ACN and 1% TFA, were subsequently eluted using a 25% ammonia solution in 40% ACN and were lyophilized and stored at −80 °C until MS measurement. Phosphopeptides were analyzed in a Q-Exactive or Q-Exactive HF instrument (Thermo Fisher Scientific, Bremen, Germany) using a 4.5 h gradient in a data-dependent Top10 MS acquisition method using one full scan (300–1750 *m/z*, MS resolution 120,000) and a set target of 3 × 10^6^ ions. The ions were fragmented with higher-energy collisional dissociation (HCD) and were analyzed by data-dependent MS/MS scans with the following parameters: a target of 2 × 10^5^ ions, a maximum ion fill time of 108 ms, an isolation window of 1.3 *m/z,* a normalized collision energy (NCE) of 28% and an MS/MS resolution of 60,000). Dynamic exclusion for 20 s was enabled to prevent the repeated sequencing of peptides.

### 2.3. MS Bioinformatics and Data Analyses

MS bioinformatics and data pre-processing were performed as described previously [45]. Principle component analysis (PCA) was performed in the Perseus environment by median averaging the phosphosites and by imputing missing values from the normal distribution (width = 0.5 and downshift = 1.5). Imputation was performed only for PCA, and for all other analyses, imputation was not performed. To identify responsive phosphosites, we separated the LD and HD data and used a combination of two criteria, with the first being having a significant response (Analysis of Variance (ANOVA; FDR < 0.05)) and the second crossing a 1.5-fold change cut-off. For the analyses presented in Figures related to wt cells, LD (ANOVA; FDR < 0.05, df = 8, F = 28,268) and HD (ANOVA; FDR < 0.05, df = 8, F = 28,206) responsive sites were identified by combining significant ANOVA sites with those that passed a 1.5-fold change in 3/6 replicates. For the analyses presented in Figures containing data from DRD cell lines, LD (ANOVA; FDR < 0.01, df = 21, F = 35,291) and HD (ANOVA; FDR < 0.01, df = 21, F = 35,231) responsive sites were identified by combining significant ANOVA sites with those that passed a 1.5-fold change in 4/6 (wt cells) or 4/4 (DRD cells) replicates. For all statistical tests, unless otherwise indicated, a permutation-based FDR was calculated and filtered to retain those with an FDR below 0.05. Cluster analysis (hierarchical clustering) was performed using z-scored data without changing the pre-set parameters of Perseus.

### 2.4. Cloud-Enabled High-Performance Computing (cHPC)

Cumulative phosphoproteome analysis (CPA) was performed in a cHPC cluster (SurfSara) as described previously [45]. Briefly, all the datasets [46,47,48] were downloaded onto the cloud server and were searched using MaxQuant in a Windows 10 virtual machine with 64 cores and 150 GB of RAM. Searches were performed by grouping SILAC samples and label-free samples as separate groups with either SILAC labels turned ON or OFF, respectively. Match between runs (MBR) was performed by carefully labelling raw files of bRP-proteome fractions, SCXPhos fractions and single-shot experiments with distinct fraction numbers to prevent matching between these experiments.

### 2.5. BrU-Seq Sample Preparation

Bru-seq experiments were carried out as published previously [49] (Paulsen et al., 2103). Briefly, bromouridine (BrU) was added to the culture media at a final concentration of 2 mM, and the dishes were returned to a CO_2_ incubator at 37 °C for 30 min to label the nascent RNA. Labeling was performed in the last 30 min of the corresponding incubation times for each experiment. Cells were trypsinized, and collected pellets were frozen in liquid nitrogen and kept frozen at −80 °C until use. From then on, the BrU-seq protocol was applied. Cell pellets were then lysed in Trizol, total RNA was isolated and BrU-RNA was immunoprecipitated using anti-BrdU antibodies (Cat#555627, BD Pharmingen, San Diego, CA, USA). We prepared strand-specific DNA libraries (Cat# RS-122-2001, TruSeq kit; Illumina, San Diego, CA, USA). Samples were then deep sequenced using Hiseq 2500 sequencers (Illumina, San Diego, CA, USA).

### 2.6. BrU-Seq Bioinformatics and Data Analysis

RNA-seq data containing strand-specific single-ended 52 bp sequencing reads were aligned to mouse ribosomal DNA complete repeating units (GenBank BK000964.1) using Bowtie (v0.12.8; parameters: n3, k1 and m1), and matching results were discarded. Unaligned reads were then mapped to the mouse genome (mm9) using TopHat (v1.4.1; parameters: min-isoform-fraction 0, max-multi-hits 1, no-closure-search, no-coverage-search, bowtie-n and initial-read-mismatches 3). We calculated Reads Per Kilobase per Megabase (RPKM) of the library size using the methods described in [50]. Briefly, a meta-transcript was created by merging all isoform exons with all mapping reads counted, and it was normalized (size of both the meta-transcript and the library). DESeq (v1.26) in R statistical software (v3.3.1) was used to determine fold-change by comparing controls and irradiated samples per time point. The data were filtered to contain only NTs that passed an FDR of 0.1, a mean RPKM ≥ 0.5 and a gene length > 300 bp. Furthermore, we removed 14 genes that passed this data filtering criterion but that had quantification values only in one experiment, thus lacking dynamic data from further data processing steps.

### 2.7. Pathway, Motif and Enrichment Analyses

The identification of enriched pathways from IR-responsive NTs was achieved by the “Expression Analysis” module of Ingenuity Pathway Analysis (IPA, Qiagen) software. To identify key transcription factors upstream of the transcriptional events, we used the “upstream analysis” module of IPA. We used a z-score of 2 (activation) and −2 (inhibition) as threshold values to call significant activation or inhibition, respectively. Significant entities (pathways and transcription factors) represent those with a Benjamini–Hochberg FDR correction value below 2 (−log 10).

We used IceLogo [51] to identify significantly (*p*-value < 0.05) overrepresented motifs. Amino acid sequences of the total phosphosites from either individual samples or the entire phosphoproteome dataset were used as background depending on the analyses performed.

For phosphoproteomes, protein-level gene ontology (GO) enrichments were performed using Reactome pathways (FDR < 0.05), and phosphosite-level pathway enrichments were performed using IPA. For nascent transcriptomes, ChIP enrichment analysis (ChEA; ver 2016) was performed using the Enrichr web tool [52], and GO analysis was performed using Reactome pathways (FDR < 0.05).

### 2.8. High-Throughput (HM) Microscopy Experiments

For mitotic index HM experiments, cells were trypsinized and collected by centrifugation (800 rpm for 8 min) in 15 mL tubes. Pellets were subsequently broken by tapping. An amount of 5 mL of cold hypotonic solution (0.4% sodium citrated (Na_3_C_6_H_5_O_7_) and 0.4% KCl) was added slowly while vortexing at 800 rpm, and the samples were incubated for 10 min at RT. Subsequently, samples were pelleted by centrifugation (800 rpm for 8 min), the supernatant was discarded, pellets were broken by tapping and cells were fixed by adding 5 mL of fixative (methanol: acetic acid at a ratio of 3:1) drop by drop (1, 2, 3, 4, 5, 6, 7, 8, 9 and 10 drops and then 0.5 mL at a time) while vortexing. Cells were then centrifuged (800 rpm for 8 min), the supernatant was discarded, and pellets were broken by tapping and were fixed two more times. Pellets were subsequently prepared for slide dropping with 10–20 drops of fixative to achieve a suitable cell density and were dropped on slides (3–4 drops per slide). Cells were then stained with DAPI for 10 min and were mounted with Aqua-Polymount. Metafer software (MetaSystems, Newton, MA, USA) was used to analyze both mitotic cells. LD and HD data were separated, and wt cells were compared with Lig4^−/−^, Xrcc5^−/−^ and PPP1R7 S12A^−/−^ clone A20. Significant differences were determined using a 2-way ANOVA and a post hoc Dunnett’s statistical hypothesis testing with a *p*-value cut-off set at <0.05 and a 95% confidence interval (CI).

For micronuclei HM experiments, cells were treated with Cytochalasin B (3 µg/mL) for 16 h to obtain bi-nucleated cells. Cells were then treated with cold hypotonic solution (5.6 g/L KCl in water) and were fixed using the fixative (methanol: acetic acid at a ratio of 4:1). All slides were stained with DAPI (1 µg/mL in PBS) for 10 min, rinsed in water, dehydrated, and embedded with City Fluor. Bi-nucleated cells were analyzed for the presence of micronuclei and scored up to 2000 binucleated cells per slide.

### 2.9. High Content-Analysis (HCA)

For high content-analysis (HCA), 60,000 cells were seeded per well in mESC medium in 96-well plates in six replicates per condition. Thirty minutes before radiation or mock-treatment, 5-Ethynyl deoxyUridine (EdU 0.5 µM) and nocodazole (100 ng/mL) were added to the medium to label S phase cells to arrest cells in the G2-M phase. After post-treatment incubation, the medium was decanted, and cells were fixed with 4% formaldehyde for 15 min. Cells were then permeabilized (0.5% Triton x−100 in blocking buffer (10% Roti immunoblock (Cat#T144.1; Carl Roth GmbH, Karlsruhe, Germany) in PBS) for 15 min, were blocked with blocking buffer for 15 min and were stored in PBS at 4 °C until staining was performed. Immunostaining was performed by incubation with either rabbit anti-53bp1 (Cat# NB100–304, Novus biologicals, Littleton, CO, USA) or rabbit anti-pS15-p53 (Cat#9284S, Cell Signaling Technology, Danvers, MA, USA)—both at a 1:1000 dilution—for 2–3 h at RT together with mouse anti-cyclin B1 (1:100 dilution; Thermo Fisher scientific, Waltham, MA, USA), two washes with blocking buffer and incubation with secondary anti-mouse Alexa Fluor 488 (A-11034) and 594 (Cat# A-11032 Thermo Fisher scientific, Waltham, MA, USA). EdU staining was performed using the Click-it EdU imaging kit (Cat# C10340, Thermo Fisher scientific, Waltham, MA, USA) according to the manufacturer’s protocol. Nuclei were stained with DAPI, and cells were stored in PBS at 4 °C until being analyzed by Cellomics (Thermo Fisher scientific, Waltham, MA, USA).

### 2.10. High-Throughput Cellular Bioenergetics (HCB)

High-throughput cellular bioenergetics (HCB) experiments were performed as published previously [53] with minor modifications for mESCs. Briefly, 100,000 cells were seeded per well (pre-coated with 0.1% gelatine) in XF medium (100 mL of RPMI-1640 medium with L-glutamine, without glucose and sodium bicarbonate; Sigma, Amsterdam, The Netherlands) supplemented with 5.5 mL FBS, 0.25 mL β-mercaptoethanol (Cat# Gibco 31350010, Thermo Fisher scientific, Waltham, MA, USA) and 11 μL leukemia inhibitory factor (LIF) (Cat# ESG1107, Millipore, Burlington, MA, USA). In all experiments, cells were plated in 180 µL XF medium, were centrifuged to obtain a monolayer and were incubated in a CO_2_-free incubator at 37 °C for 1.5 h before measurements. The following chemicals were added to the drug ports of the cartridge to perform four different conditions per well: 20 μL of 100 mM D (+)Glucose (100 g/L = 0.55 M; (Cat# G8644-100 mL, Sigma, Amsterdam, The Netherlands)), 22 μL of 10 μM Oligomycin A 1 mg (Cat# 11342, Cayman Chemical, Ann Arbor, MI, USA)) and 25 μL of 30 μM carbonyl cyanide 4-(trifluoromethoxy) phenylhydrazone (FCCP, (Cat# C2920-10 MG, Sigma, Amsterdam, The Netherlands)) to ports A, B and C, respectively. In port D, 28 μL of 10 μM Rotenone 1 g (Cat# R8875-1 G, Sigma, Amsterdam, The Netherlands) and 28 μL of 10 μM Antimycin A 25 mg (Cat# A8674-25 MG, Sigma, Amsterdam, The Netherlands) were added. The chemicals were injected automatically through the drug ports at different time points, and bioenergetics measurements were performed for different time points. Extracellular acidification rates (ECAR) were measured with a metabolic flux analyzer (96 XP; Seahorse Bioscience, North Billerica, MA, USA). Data were visualized, inspected and quality controlled using wave software (v2.6.0) and were exported to Prism (GraphPad, v7.02) for further downstream analyses. Significance was determined by performing a one-sample t-test to compare the medians of ECAR/OCR measurements to a hypothetical value of 100 with a significance level (alpha) set at 0.05.

### 2.11. Proliferation Assays 

Long-term proliferation experiments were performed by repeatedly plating 5 million cells per 10 cm dish. After plating, cells were irradiated and allowed to grow for 24 h. Cells were counted using a Coulter counter, and the population doubling time was determined.

For determining subtle differences in proliferation rates, IB10 wt, *Polq*^−/−^, *Lig4*^−/−^ and *Xrcc5*^−/−^ cells were seeded in an equal ratio (1:1:1:1), and long-term proliferation experiments were performed as described above. Restriction fragment length polymorphism (RFLP) assays were carried out as described previously [42]. Briefly, cell pellets were collected at every passage of proliferation experiments and were kept frozen at −80 °C until use. DNA was isolated from the mixed population of *Polq*^−/−^, *Lig4*^−/−^ and *Xrcc5*^−/−^ cells, and PCR analysis was performed. Cell lines were identified based on the loss of a unique restriction site in the knockout clones (Figure S4D). Proportions of *Polq*^−/−^, *Lig4*^−/−^ and *Xrcc5*^−/−^ cells in the mixed cell population over time were calculated using Image Studio Lite Ver 5.2 (LI-COR Biosciences, Lincoln, NE, USA) (Figure S4C).

### 2.12. Gene Editing Using CRISPR-Cas9

For CRISPR/Cas9-mediated phosphosite mutations of PPP1R7, two oligos of sgRNAs were cloned into pX458 plasmids by annealing, phosphorylation, and ligation reactions. The plasmids containing sgRNAs were subsequently transformed into Dh5alpha, and the plasmids were isolated using a miniprep kit and were verified by sequencing. Briefly, 8 million cells were transfected in suspension after trypsinization for 30 min at 37 °C and were subsequently seeded onto gelatine-coated dishes. Cell transfections were performed by mixing 700 µL Opti-MEM medium, 42 µL Lipofectamine 3000 (3:1 lipofectamine: DNA ratio; Cat# 15282465, Invitrogen, Waltham, MA, USA), 28 µL p3000-reagent and 14 µg DNA (8 µg ssODN, 3 µg plasmid containing sgRNA and 3 µg Rad51 cDNA (MGC mouse Rad51 cDNA CloneId:4951015) containing plasmid (glycerol stock: Cat# MMM1013-202767140, Dharmacon, Lafayette, CO, USA)). Sequences of ssODN and sgRNA are shown in Figure S3J. After transfection, cells were sorted by flow cytometry (top 10–15% of the parent GFP expressing cells) to select cells that contained pX458 plasmid; GFP positive cells were seeded at 4000 cells per 10 cm dish to pick colonies to grow in 96-well plates. Each clone was subsequently sequenced to determine the mutation. 

## 3. Results

### 3.1. High-Temporal-Resolution Map of the Dose-Dependent Phosphoproteome after IR

**Figure 1:**
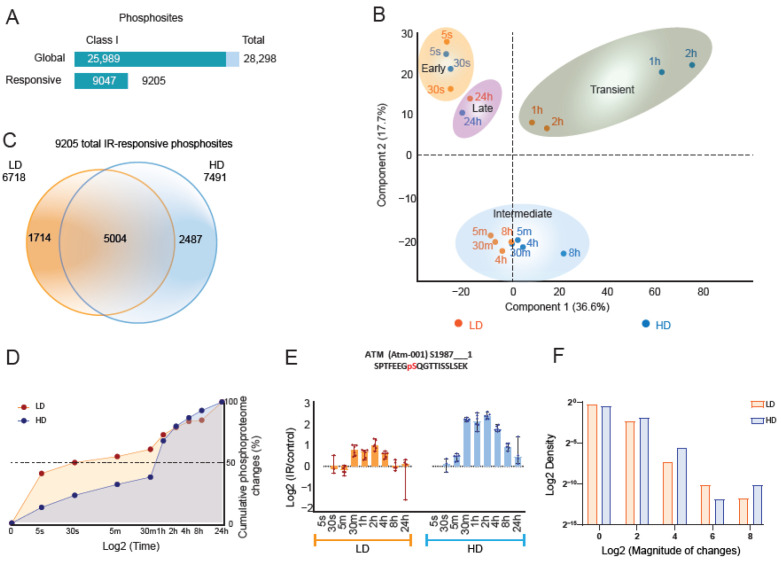
IR dose-dependent phosphoproteome dynamics: (**A**) Statistics of phosphosites quantified in IB10 mouse ES cells. Phosphosites quantified and those that responded to DNA damage are depicted in the top and bottom bars, respectively. Light bar indicates total phosphosites and the dark bar indicates class I phosphosites (>0.75 phosphosite localization probability). (**B**) Principal component analysis (PCA) map of log2 SILAC ratios of total responsive phosphosites after LD (orange dots) and HD (blue dots) exposure over unirradiated controls. Four groups are readily visible. (**C**) Venn diagram of 9205 phosphosites that responded to LD (orange circle) and HD (blue circle). (**D**) Temporal dynamics showing the timing of phosphoproteome changes in LD- and HD-exposed mESCs. (**E**) Bar charts showing quantifications of ATM phosphosite pS1987 in response to LD and HD. Dots and bars represent quantifications and median values of up to six replicates, respectively, and error bars represent a 95% confidence interval (CI). (**F**) Density plot showing the distribution of the magnitude of global phosphoproteome changes in LD- and HD-exposed cells.

To determine the temporal dynamics of global phosphoproteome changes after LD and HD, as a part of our systems analysis (Figures S1 and S2), we irradiated SILAC-labeled wild-type (wt) mESCs and collected cell lysates at nine different time points. We profiled the phosphoproteomes using a high-throughput phosphoproteomics (HighPhos) method [45] in six biological replicates per condition (Figure S1D) and quantified a total of 28,298 distinct phosphosites (25,989 Class I phosphosites) (Figure 1A).

Principal component analysis (PCA) of HighPhos datasets segregated the dataset into four groups: early (5 s and 30 s), intermediate (5 m, 30 m, 4 h, and 8 h), transient (1 h and 2 h) and late (24 h) responsive groups. The transient and intermediate groups further showed dose-dependent segregation (Figure 1B). In total, 9205 phosphosites (in 2395 proteins) were IR-responsive, of which 5153 (~56%) contained two or more phosphorylated amino acid residues. A total of 1714 (on 938 proteins) and 2487 (on 1196 proteins) phosphosites responded specifically to LD and HD, respectively (Figure 1C), and 5004 phosphosites (on 1604 proteins) responded to both LD and HD, revealing high similarity to the extent of phosphoproteome responses to LD and HD. The pathway analysis of phosphoproteins that contained LD-specific, HD-specific or shared phosphosites uncovered numerous terms related to the cell cycle, DNA damage response (DDR) and apoptosis without any dose-specific terms (Supplementary Table S1).

The experimental setup with six biological replicate exposures allowed us to quantify very early global phosphoproteome changes after LD and HD. Although about half of the phosphoproteome changes occurred within 30 s after LD exposure, it took more than 30 min to reach this proportion after HD exposure. However, irrespective of the applied dose, about 80% of the phosphoproteome changes occurred within the first two hours after IR (Figure 1D). The phosphorylation states of regulatory phosphosites of DDR kinases, including ERK1/2, mTOR and p38MAPK, were altered almost instantaneously (5 and 30 s) after both LD and HD (Figure S2D). Although several DNA-damage-sensing proteins, including ATM, have been reported to be activated within seconds after irradiation [54,55], the enhanced auto-phosphorylation of S1987 of ATM was not observed at those early time points either after LD or HD (Figure 1E). Moreover, differences in phosphorylation amplitude between LD and HD were not manifest for most of the phosphorylation changes over the entire post-radiation time period (Figure 1F). A dose of 0.1 Gy was sufficient for most phosphorylation events to reach their maximal magnitude, indicating that dose-dependent phosphorylation events after IR-exposure are relatively rare. Additionally, the temporal dynamics of the global phosphoproteome response to LD and HD appeared to be highly similar, as shown by unsupervised hierarchical clustering of the 9205 IR-responsive phosphosites (Figure S2E,F).

**Figure 2:**
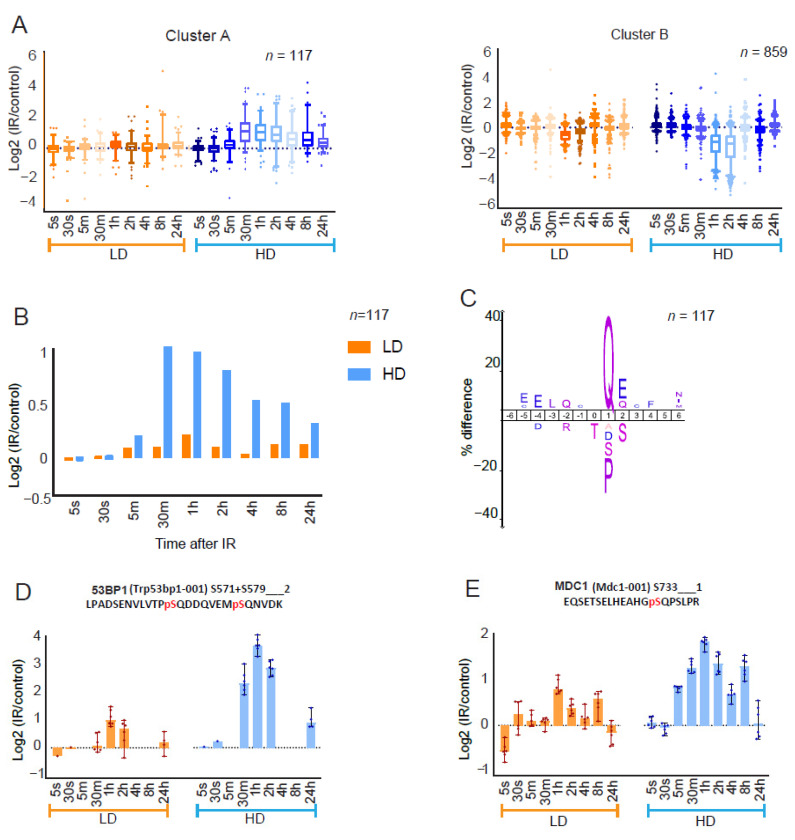

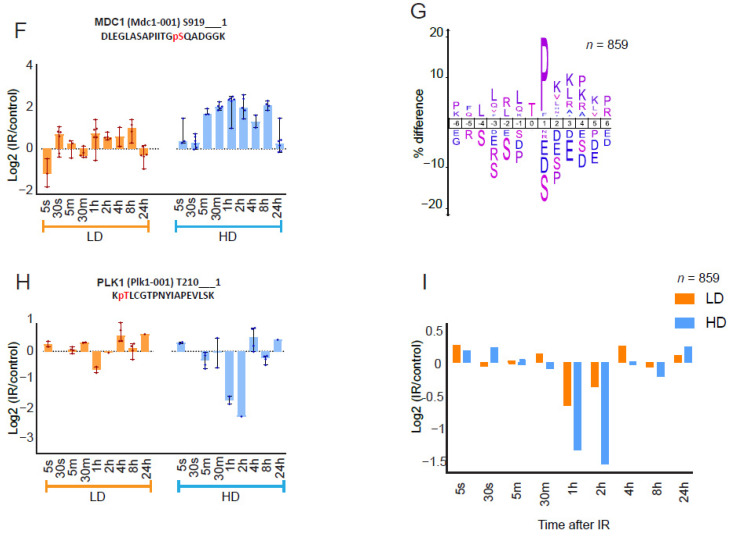
Dynamics of DDR signaling cascades: (**A**) Box and whisker plots of log2 SILAC ratios of averaged phosphosites of two different row clusters (hierarchical clustering) that show IR-dose dependent amplitude differences. Orange and blue plots indicate LD and HD data points (median value of six replicates for each phosphosite), respectively. Whiskers represent the 5–95 percentile. (**B**) Sequence motif (IceLogo) of cluster A. The phosphorylated amino acid is located at position 0. (**C**) Bar chart of relative median phosphorylation of 117 cluster A phosphosites after LD (orange) and HD (blue). (**D**–**F**) Dynamics of 53BP1 (pS571 + pS579) and MDC1 (pS733 and pS919), respectively. (**G**) Cluster B sequence motif. (**H**) Dynamics of PLK1 T210 dephosphorylation in response to LD and HD. (**I**) Bar chart of relative median phosphorylation of 859 cluster B phosphosites after LD (orange) and HD (blue).

We screened the datasets for clusters that showed dose-dependency in their dynamic profiles and uncovered two clusters (A and B) that displayed amplitude differences between LD and HD (Figure 2A and Figure S2E,F). Median phosphorylation amplitudes of cluster A (117 phosphosites on 71 proteins) that included ATM pS1987 (Figure 1E) were significantly higher for HD than those of LD, although the phosphorylation kinetics were similar (Figure 2B). The pathway analysis showed that proteins in cluster A were enriched for DDR terms (Figure S2G) with an enrichment for the (S/T)Q motif among phosphosites (Figure 2C). Consistently, we observed dose-dependent enhanced phosphorylation of several ATM substrates, including 53BP1 (pS571 + pS579) (Figure 2D), BRCA1 (pS1422) (Figure S2H) and MDC1 (pS592, pS733 and pS919) (Figure 2E,F and Figure S2I) as well as of replication-related proteins MCM3 (pS732, a regulatory site, and pS738) and MCM6 (pS704) (Figure S2J). Other previously described ATM-dependent phosphosites in this cluster include MDC1(pS168), SMC1A(pS360), ARID1A(pS1184), DCK (pS74) and HMGA1(pS44) [27,31]. Cluster A encompasses non-(S/T) Q phosphosites such as MDC1 (pS168 + pS176) and CHEK2 (pS264 and pS265), which represent potential substrates of ATM-activated downstream kinases [31] (Figure S2K). Moreover, we found indications for replication-stress-related signaling involving ATR. Activated ATR promotes CHEK1-mediated checkpoint signaling and FANCI-FANCD2-mediated signaling upon stringent and moderate levels of replication fork slowing, respectively [56,57,58]. Although CHEK1 S317 showed limited phosphorylation after both LD and HD with kinetics and an amplitude different from those of cluster A phosphosites (Figure S2L), FANCI (pS554 + pS555 and pS555 + pT558) phosphorylation was enhanced transiently (1 h) after LD, and it persisted (0.5–24 h) after HD exposure (Figure S2M).

Cluster B (859 phosphosites on 401 proteins) displayed dose-dependent dephosphorylation amplitudes at 1 h after LD and 1–2 h after HD (Figure 2A). Proteins in this cluster were enriched for cell cycle terms (Figure S2N), with phosphosites being overrepresented for the (S/T) P motif (Figure 2G) as well as for threonine residues, representing the preferred targets of mitotic kinases and phosphatases, respectively [59,60]. Mitotic kinases (such as CDK, NEK, PLK, MAPK and Aurora) and phosphatases (such as Cdc14, PP1 and PP2A) temporally order cell cycle phosphorylation in a coordinated manner, thereby controlling mitotic entry and exit, respectively [61,62]. We observed dose-dependent dephosphorylation dynamics in two sites in polo-like kinase1 (PLK1), including the activation site (pT210) (Figure 2H and Figure S2O) and a site (pS712) in the cell division cycle-associated protein 2 (CDCA2; Repo-Man)—a regulator of mitotic phosphatase PP1 (Figure S2P). Additionally, several phosphosites of three “core” mitotic Aurora kinase co-factors were dephosphorylated in a dose-dependent manner: BORA (pS370 + pS372), INCENP (pS273, pS284 and pS762 + pT771) and TPX2 (pT113, pS121 + pS125, pT369 and pS737) (Figure S2Q,S). Several phosphosites of substrates of CDK (e.g., LMNA pS390 + pS392) and MAPK (e.g., MYBBP1A pT1277 + pS1280) were also part of cluster B (Figure S2T,U). These results provide evidence for transient dephosphorylation-dependent inactivation of the mitotic signaling cascades, including the CDK–MAPK and Aurora–PLK1 pathways [63]. The global kinetic plot of cluster B revealed similar dephosphorylation kinetics after LD and HD (Figure 2I).

### 3.2. DSB Repair, Signaling Dynamics and G2/M Checkpoint Activation after LD and HD

**Figure 3:**
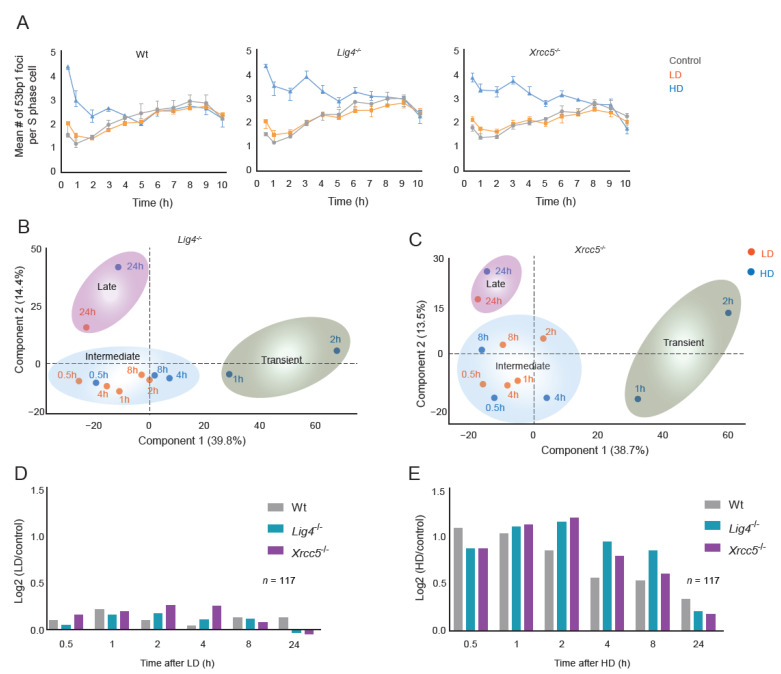
Dynamics of DDR signaling cascades in DSB repair-deficient cells: (**A**) Dynamics of 53BP1 foci decay in wt, Lig4^−/−^ and Xrcc5^−/−^ S-phase cells. Dots and error bars represent the median values of six replicates and 95% CI values, respectively. (**B**,**C**) PCA maps of log2 SILAC ratios for Lig4^−/−^ and Xrcc5^−/−^ cells. (**D**,**E**) Bar chart of relative median phosphorylation of cluster A phosphosites after LD and HD in wt and DRD cells.

**Figure 4:**
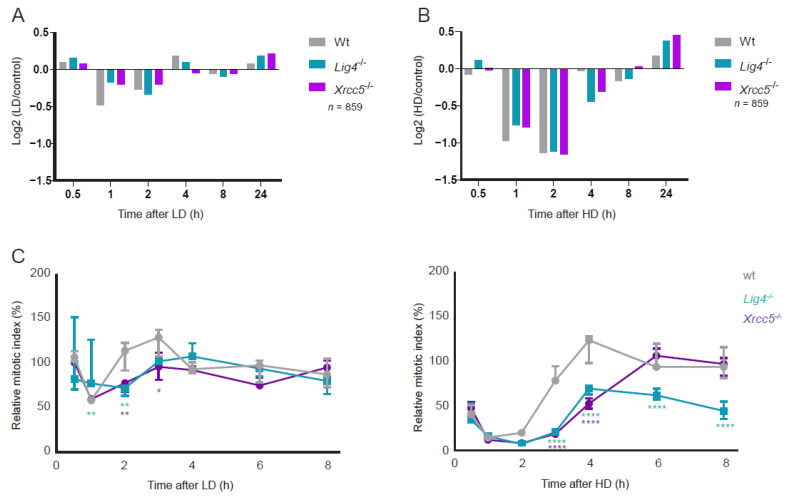
G2/M checkpoint dynamics: (**A**,**B**) Bar chart of relative median phosphorylation of 859 cluster B phosphosites after LD and HD in wt and DRD cells. (**C**) Relative mitotic index (in % of metaphases in IR-exposed cells over mock-exposed cells) of wt, Lig4^−/−^ and Xrcc5^−/−^ cells in response to LD (left panel) and HD (right panel). Statistics: two-way ANOVA with a post hoc Dunnett’s test at 95% CI between wt and Lig4^−/−^ or Xrcc5^−/−^. (* *p*-value < 0.05, ** < 0.01 and **** < 0.0001).

To investigate how IR-induced signaling dynamics were affected by the prolonged presence of DNA damage, particularly DSBs, we quantified the phosphoproteomes of two DNA damage repair-deficient (DRD) cell lines that were defective in non-homologous end joining, i.e., Lig4^−/−^ and Xrcc5^−/−^ (Ku80) after LD and HD (Figure S1E). Analyses of IR-induced 53BP1 foci in S- and non-S-phase cells confirmed the impairment of DSB repair in these cells (Figure 3A and Figure S3A,D). Temporal dynamics of the phosphoproteomes of wt and DRD cells revealed, in total, 35,358 quantified phosphosites across 306 biologically independent experiments. These included over 1300 (S/T)Q phosphosites, of which 321 were exclusively quantified in DRD cells. Although PCA analysis segregated the phosphoproteomes of DRD cells after HD (Figure 3B,C) like wt cells (Figure 1B), the transient and intermediate groups did not segregate after LD, indicating altered signaling dynamics. We identified by stringent statistical testing 3906 phosphosites that significantly responded to LD or HD, and we performed hierarchical cluster analysis. Compared with wt cells, clusters A and B in DRD cells displayed sustained (de)phosphorylation kinetics with a delayed signal peak after LD and HD (Figure 3D,E), whereas the amplitudes of clusters A and B only marginally increased (cluster A) or decreased (cluster B) in DRD cells (Figure 3D,E and Figure 4A,B). Thus, it appears that the impaired repair of DSBs adjusts the proportionality of cellular responses governed by clusters A and B mainly by altering the kinetics rather than the amplitude of phosphorylation. Nevertheless, it is obvious—particularly after HD—that phospho-signaling in wt and DRD cells continues at times when no excess 53BP1 foci are observed, and DSB repair appears to be completed (Figure 3A,E). Furthermore, we observed that some phosphosites, such as those in ATM (pS1987) and MDC1 (pS733), showed no distinct kinetic differences between irradiated DRD and wt cells after both LD and HD, whereas the phosphorylation amplitude of other phosphosites (such as MDC1 pS919) was elevated in DRD cells (Figure S3E). Moreover, replication stress-related ATR-FANCI signaling was activated in both wt and DRD cells after HD exposure, whereas ATR-CHEK1 signaling appeared to be activated in DRD cells and to a lesser extent in wt cells (Figure S3F).

In LD-exposed DRD cells, the dephosphorylation of cluster B proteins was reduced in amplitude at 1 h after radiation compared with wt cells (Figure 4A). HD-exposed DRD cells displayed early dephosphorylation of cluster B proteins at similar levels as wt cells but showed delayed recovery of dephosphorylation, most notably at four hours (Figure 4B). Since protein (de)phosphorylation is crucial for cell cycle control, we profiled the G2/M-checkpoint dynamics following LD and HD (Figure S1H). In wt cells, the mitotic index was reduced by 40% and 90% 1 h after LD and HD with complete recovery within 2 h and 4 h, respectively (Figure 4C). Installment of the G2/M arrest occurred with similar dynamics in DRD cells as in wt cells, but its recovery was delayed. After LD, the mitotic index returned to the level of un-irradiated cells by 4 h in both DRD cell lines. After HD, the G2/M checkpoint recovery in Xrcc5^−/−^ cells was complete at 6 h, whereas Lig4^−/−^ cells had not completely recovered at 8 h post-irradiation. The observed difference in the rate of G2/M checkpoint recovery is in line with the prolonged cluster A signaling after HD in Lig4^−/−^ compared with Xrcc5^−/−^ cells (Figure 3E).

These results indicate that impaired DSB repair alters the dynamic properties of DDR signaling cascades and that G2/M checkpoint recovery is independent of DSB repair completion.

### 3.3. Multi-Site Dephosphorylation of Cell Cycle Proteins Activates the G2/M Checkpoint in mESC

Mitotic entry is irreversible due to a balance between inhibitory and activating cellular mechanisms that function as a bistable switch controlling the pivotal mitotic entry regulator CDK1-cyclin B [64] and the crucial mitotic exit regulator phosphatase PP2A: B55 [65]. Bistable switches are an emergent cell signaling feature conferred by ultrasensitivity, i.e., a dramatic cellular response like mitosis upon small changes such as multi-site phosphorylations [66]. We probed for the existence of multi-site (de)phosphorylation and observed a higher proportion of double/multiple sites (Figure S3G) among dephosphorylated proteins in cluster B (64%, enriched for cell cycle proteins, Figure S2N) following IR when compared with cluster A (38%, enriched for DDR proteins, Figure S2G). Conversely, and in support of this, motif analysis on the 3906 IR-responsive phosphosites identified from the wild-type (wt) and two mutant mESCs defective in non-homologous end joining (NHEJ), i.e., Lig4^−/−^ and Xrcc5^−/−^ (Ku80), after stringent statistical testing (see below), uncovered double/multi-site phosphorylation enriched specifically for a proline-directed motif, (S/T)P (Figure S3H,I), representing a substrate motif of cell cycle kinases [67]. The proportion of single and multi-site phosphorylation events between LD and HD irradiation was not significantly different.

### 3.4. Phospho-Signaling via PPP1R7-S12 Is Involved in G2/M Checkpoint Activation

**Figure 5:**
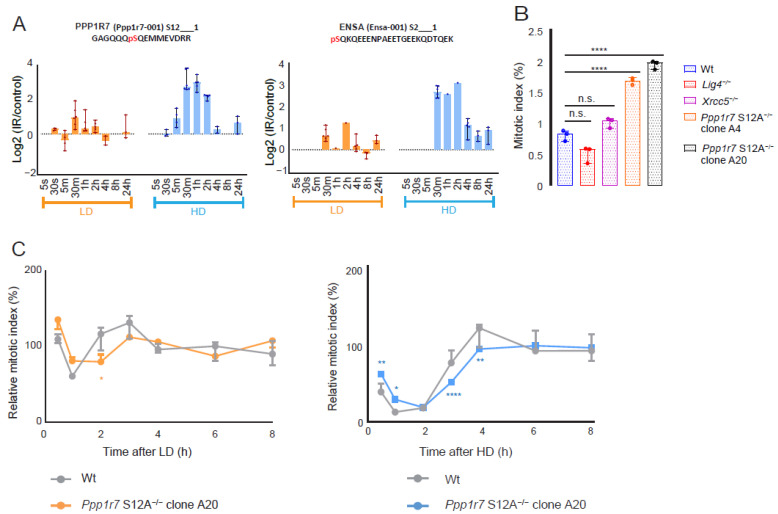
Phospho-S12 PPP1R7 signaling dynamics, mitotic exit, and checkpoint activation in mESCs: (**A**) Dynamics of PPP1R7 pS12 and ENSA pS2 in response to LD and HD. (**B**) Mitotic index (in % of metaphase cells among total population in the absence of exogenous DNA damage) of five different cell lines: wt, Lig4^−/−^, Xrcc5^−/−^, Ppp1r7 S12A^−/−^ (clone A4) and Ppp1r7 S12A^−/−^ (clone A20) cells. Statistics: one-way ANOVA test between wt and other cell lines. (**** *p*-value < 0.001; n.s. = not significant). (**C**) A kinetic plot of the relative mitotic index of wt (in grey; data are taken from Figure 4C) and clone A20 cells in response to LD (in orange) and HD (in blue). Dots and error bars represent median and 95% CI values, respectively. Statistics: two-way ANOVA with a post hoc Dunnett’s test at 95% CI between wt and clone A20. (* *p*-value < 0.05, ** < 0.01 and **** < 0.0001).

Phosphatase PP1, one of the seven members of the phosphoprotein phosphatase (PPP) family, has been proposed to initiate the mitotic exit regulator PP2A: B55 [68,69]. PPP1R7 (SDS22) and ENSA are critical regulators of PP1 and PP2A: B55, respectively, and both are essential for the proper completion of mitosis. PPP1R7 targets PP1 to the mitotic kinetochore [70], whereas phosphorylated ENSA is a specific inhibitor of PP2A: B55 during mitotic entry [71]. Both PPP1R7 and ENSA are present in cluster A and displayed dose-dependent enhanced phosphorylation in their sole SQ motif (i.e., PPP1R7 pS12 and ENSA pS2) (Figure 5A). We hypothesized that the S12 phosphorylation of PPP1R7 might be critical for the rapid activation and/or recovery of a G2/M checkpoint arrest upon DNA damage infliction. Using CRISPR-Cas9 gene editing, we derived two homozygous PPP1R7 mutant clones, in which the S12 of PPP1R7 was altered into non-phosphorylatable alanine (PPP1R7-A12) (Figure S3J). Unexpectedly—even in the absence of exogenous DNA damage—the mitotic index of these mutant clones was significantly enhanced (1.83%) compared with that of wt (0.82%) and NHEJ-deficient DRD (Lig4^−/−^ 0.52%; Xrcc5^−/−^ 1.02%) cells (Figure 5B). After irradiation, the activation of a G2/M checkpoint and its recovery were significantly delayed in the PPP1R7-A12 mutants in a dose-dependent manner (Figure 5C). Together, these results suggest that the loss of PPP1R7-S12 phosphorylation diminishes mitotic exit control in undamaged cells and delays the activation of the G2/M checkpoint and checkpoint recovery following IR-induced DNA damage.

### 3.5. Proliferation Responses after LD and HD

The installment of a G2/M arrest after IR exposure implicates a reduction in the proliferation rate. Following seven repeated exposures, each separated by 48 h, we observed only a subtle reduction in proliferation capacity after LD exposures in DRD cells (Figure S4A), whereas a prominent reduction in proliferation capacity was observed after HD exposure, especially in DRD cells (Figure S4B). To measure subtle changes in cell proliferation, we designed a sensitive competition assay in which wt, Lig4^−/−^ and Xrcc5^−/−^ cells, as well as Polq^−/−^ cells (deficient in DNA polymerase theta mediated end joining), were mixed in equal ratios (1:1:1:1). Cell population composition changes were determined by restriction fragment length polymorphism analyses. A reduction in the proportion of Lig4^−/−^ and Xrcc5^−/−^ cells became apparent following LD exposure (Figure S4C,D), consistent with their delayed G2/M checkpoint recovery. The proliferation of Polq^−/−^ cells was only marginally affected by LD, consistent with their slightly enhanced sensitivity to high IR doses [42]. Inherent to the method, the competition assay does not allow the detection of subtle changes in the proliferation rate of wt cells after LD.

### 3.6. Micronuclei Induction after LD Exposure

**Figure 6:**
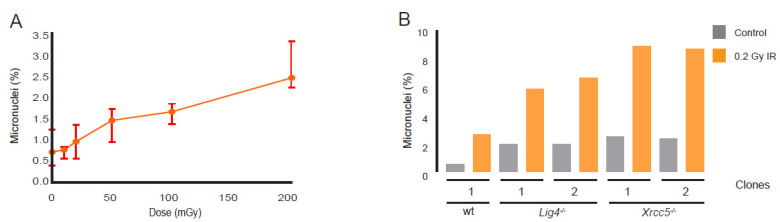
Micronuclei frequency in DSB repair-proficient and -deficient mESCs. (**A**) Induction of micronuclei (in % of micronuclei among bi-nucleated cells) as a measure of genetic damage in repair-proficient cells in response to increasing doses of IR. Dots and error bars represent median and 95% CI values, respectively. (**B**) MN frequency in wt, Lig4^−/−^ and Xrcc5^−/−^ cells in response to IR (0.2 Gy).

The activation of the G2/M checkpoint provides cells with a time window to complete DSB repair before progressing into mitosis, preventing genomic instability. The mitotic entrance of cells with unrepaired DSBs after G2/M checkpoint recovery results in the formation of micronuclei. Consistently, micronuclei formation in wt cells increased linearly with IR dose (0.01–0.2 Gy) without a distinct threshold in this dose range (Figure 6A). Micronuclei frequencies substantially increased if DSB repair by NHEJ was abrogated (Figure 6B), indicating that extension of the G2/M checkpoint in DRD cells was insufficient for alternative DSB repair pathways to prevent the progression of (late G2) cells with unrepaired breaks into the M phase.

### 3.7. Impaired DSB Repair Dynamically Rewires Crucial Signaling Pathways after IR

Unsupervised hierarchical clustering unveiled two additional clusters (C and D) marked by phosphorylation amplitude differences between DRD and wt cells (Figure S5A). Cluster C (1096 phosphosites on 580 proteins) showed an equal increase in phosphorylation amplitude in LD- and HD-irradiated DRD cells and was enriched for apoptosis, cell cycle, RNA metabolism, proliferation and MAPK/ERK signaling pathway terms (Figure S5B). The MAPK/ERK pathway integrates external signals from mitogens, thereby regulating proliferation. ERK1/2 kinase is known to activate the mTORC1 pathway [72], and accordingly, cluster C contained the mTOR kinase (autophosphorylated at S2481; activation site) and various bonafide and putative substrates of mTOR (Figure S6) as well as of ERK1/2 substrates (Figure S5C), the latter of which responded with temporal dynamics identical to mTOR and its substrates (Figure S6). Cluster D (1204 phosphosites in 573 proteins) displayed decreased phosphorylation amplitudes in irradiated DRD cells compared with wt cells and was overrepresented for apoptosis, cell cycle, RNA metabolism and mTOR signaling terms (Figure S5D). The activation of mTOR in irradiated DRD cells goes together with the persistent reduction in phosphorylation amplitudes of the negative regulator of mTOR, with GRB10 (pS429) (Figure S5E) precluding the inhibition of the mTORC1-controlled phosphatidylinositol 3-kinase (PI3K) and MAPK/ERK pathways that regulate proliferation and differentiation [73]. Likewise, the decrease in phosphorylation amplitudes of mTOR substrate ribosomal protein S6 (pS235 and pS236) (Figure S6) and ERK1/2 substrate ribosomal S6 kinase RPS6KA/p90RSK (pS352; activation site) (Figure S5E) in DRD cells may promote differentiation and the loss of pluripotency, as described for mESCs [74]. Collectively, these results suggest that the irradiation of DSB repair-deficient mESCs resulted in the sustained activation of mTOR and MAPK/ERK signaling pathways with no temporal and quantitative differences between low- and high-dose IR.

In contrast to DRD cells, DSB repair-proficient cells displayed the dephosphorylation of mTOR kinase (T2474 + S2478) and its substrates (cluster C and D, Figure S6) after LD and HD, suggesting inactivation of the mTOR pathway, although the mTOR negative regulator GRB10 only displayed enhanced phosphorylation amplitudes at 24 h (cluster D, Figure S6). We note here that, at least in WT cells, most changes post-IR were not expected to be related to changes in protein abundance levels, as we observed in a separate study in which, at 0.5 h and 4 h, only a few proteins displayed IR-induced changes at both the protein and phosphorylation levels [75]. Cluster C also disclosed subtle temporal differences between LD and HD in the quantity of doubly phosphorylated MYC/MYCN (T58 + S62) (Figures S6 and S5F), which represents a signal for its ubiquitin-mediated degradation [76,77,78]. Early after HD irradiation (5 min), cells responded with the dephosphorylation of cMYC/MYCN (T58 + S62), followed by a two-fold increase in amplitude during the next 2 h. In contrast, the extent of the phosphorylation of MYC/MYCN (T58 + S62) in LD-irradiated wt cells was unaltered during the first hour post-IR.

### 3.8. Temporal Dynamics of Gene Expression, and Metabolic Control Programs after LD and HD

**Figure 7:**
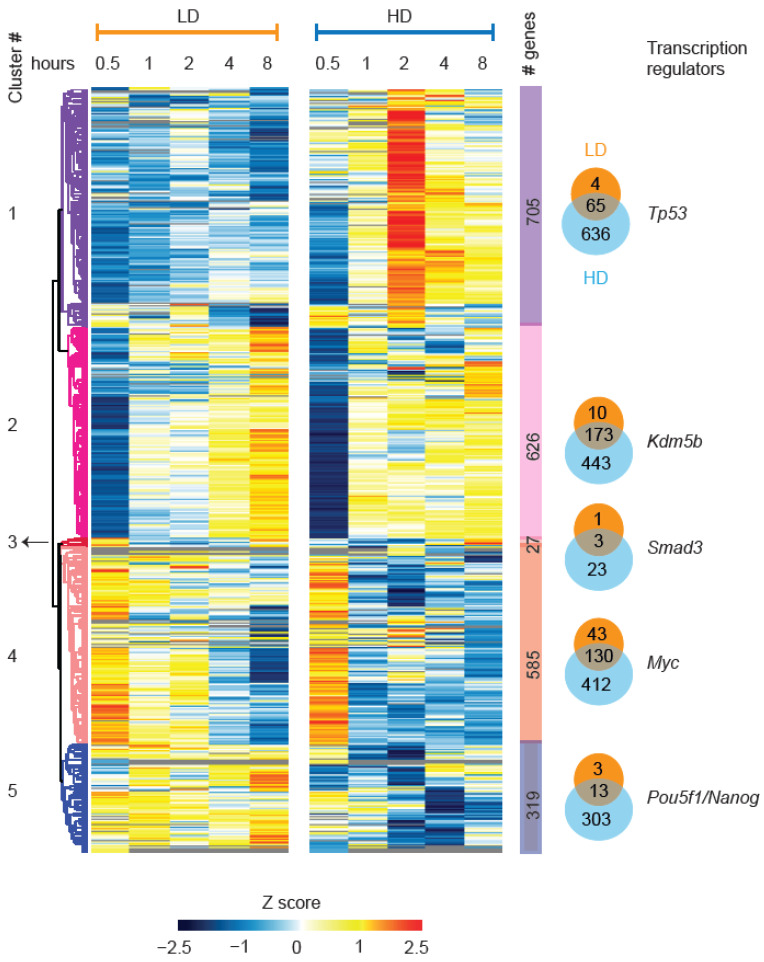
IR dose-dependent nascent transcriptome dynamics. Heatmap of hierarchically clustered z-scored log2 ratios of IR-responsive nascent transcripts (NTs). The left and right panels represent NT dynamics from 0.5 h until 8 h after LD and HD, respectively. Clusters are labeled on the left side, and the number of genes represented by each cluster is indicated on the right side inside color-coded bars. Overlap of genes between LD and HD per cluster is indicated in a Venn diagram plot, where orange and blue circles represent LD- and HD-responsive genes, respectively. The top transcription regulator enriched for each cluster using Enrichr is indicated next to the Venn diagram.

To assess the transcriptional consequences of LD- and HD-induced phosphorylation events, we performed an in-depth global analysis of nascent transcriptome dynamics in wt mESCs using Bru-seq [49]. The results revealed that 384 genes responded to both LD and HD, and 61 and 1817 genes responded specifically to LD and HD, respectively (FDR < 0.1; Figure S7A). Temporal plots of global cumulative changes to the nascent transcriptome revealed that over 80% of the LD-induced transcriptome changes occurred already within the first 30 min, and it took two hours to reach the same proportion of HD-induced changes (Figure S7B). Additionally, similar to the phosphoproteome, no differences in magnitude were observed for the vast majority of the nascent transcriptome changes after LD and HD (Figure S7C). Hierarchical clustering of the 2262 (~25%) IR-responsive nascent transcripts (NTs) uncovered five clusters (Figure 7) with distinct dynamic transcriptional changes following LD and HD (Figure S7D). An in-silico transcription regulator (TR) analysis revealed that clusters 1, 2, 3, 4 and 5 showed enrichment for p53, KDM5B, SMAD3, MYC and POU5F1(OCT3/OCT4)/NANOG, respectively (Figure 7 and Figure S7E). The pathway analysis of LD- and HD- responsive transcripts revealed dose-specific pathway activation. LD exposure resulted in the preferential activation of EIF2 and NRF2 pathways and in the inhibition of the AMPK pathway. Conversely, HD exposure specifically activated the p53 (possibly through the enhanced expression of MDM2 inhibitor CDKN2A) and senescent pathways and inhibited EIF2, sirtuin signaling, insulin receptor signaling and the pluripotency pathways (Figure S7F,G).

Multiple p53 target genes (cluster 1), including those involved in cell cycle arrest and DSB repair, exhibited a dose-dependent enhancement of transcription amplitudes, i.e., they were enhanced after HD when compared with LD (Figure S7H,I), indicating dose-dependent activation of the core DDR pathways. Transcripts in clusters 4 and 5 were activated and inhibited by LD and HD, respectively, and they comprised *Mycn, Nanog, Pou5f1 (Oct3/4)* and several of their target genes (Figure S7J). Additionally, we found that the transcripts of genes involved in glycolysis (*Gapdh, Eno1, G6pdx, Pfkp* and *Slc2a3*) [79] (Figure S7K), several histones (Figure S7L) and proto-oncogenes (*Junb* and *Pim3*; Figure S7M) were elevated after LD but reduced after HD. Given the differential expression of these glycolytic genes, we examined glycolytic flux following LD and HD. Measuring the lactate production by quantifying the relative extracellular acidification rates (ECARs) at corresponding time points confirmed increased and reduced glycolysis after LD and HD, respectively (Figure S7N). Increased levels of circulating lactate after LD might regulate and maintain mESC pluripotency by increasing intracellular α-ketoglutarate [80]. Indeed, NTs enriched for pluripotency genes (cluster 5) were activated after LD and were inhibited after HD (Figure 7). Additionally, reactive oxygen species (ROS) possibly resulting from activated glycolysis [81] may account for the NRF2-mediated oxidative stress response after LD (Figure S7F), the distinct LD-specific kinetics of several antioxidant genes (*Atf4, Sqstm1, Eef2, Srxn1, Hmox1, Pycr2*, *Cdk8, Gpx4, Prdx4* and *Prdx6*) and the transcriptional up-regulation of the major base excision repair-related apurinic/apyrimidinic (AP)-endodeoxyribonuclease 1 (Apex1; Figure S7O) [82]. Additionally, several mitochondrial genes (*Ucp2, Uqcr11, Mrpl12, Tomm5, Fkbp4, Mrpl53* and *Tomm40*; Figure S7P) and metabolic genes (*Fasn* and *Ndufs7*; Figure S7Q) displayed LD-specific enhanced expression between 0.5 h and 4 h after exposure to IR. High doses of IR are also known to activate the canonical Wnt pathway [83], a critical regulator of stem cells [84]. Genes involved in the Wnt-β-catenin signaling pathway and Wnt target genes involved in protein synthesis, including all four ubiquitin-encoding genes (*Uba52, Ubb, Ubc* and *Rps27a*) that regulate ubiquitination and proteasome activities in response to oxidative stress [85], were up-regulated after LD and down-regulated after HD (Figure S7R). Collectively, these results provide evidence for distinct transcriptional, phospho(proteomic) and metabolic responses after LD and HD that regulate anabolic metabolism, oxidative stress response, mitochondrial function, pluripotency and proliferation (LD), as well as cell cycle checkpoint activation and DSB repair (HD).

## 4. Discussion

Uncertainty on increased cancer risk from exposure to LD ionizing radiation calculated from a linear no-threshold model [6] is mainly due to insufficient mechanistic understanding of the effects of LD, especially in stem cells that are generally regarded as cells-of-origin for radiation-induced carcinogenesis [15]. To unravel the molecular mechanisms underlying the cellular response to acute low (LD; 0.1 Gy) and high (HD; 1 Gy) doses of X-rays, we performed in-depth phosphoproteome and nascent transcriptome analyses of mouse embryonic stem cells with a high temporal resolution and assessed the ensuing dynamic changes in cellular responses.

### 4.1. Phosphoproteome and Transcriptome Responses after LD and HD

The global phosphoproteome analysis of LD and HD-exposed mESCs revealed a considerable similarity to the extent of protein phosphorylation. Of the total 9205 IR-responsive phosphosites, 5004 responded to both LD and HD, and 1714 and 2487 responded specifically to LD and HD, respectively. The pathway analysis of phosphoproteins that contained LD-specific, HD-specific, or shared phosphosites did not reveal any dose-specific pathways, suggesting that differences primarily exist in phospho-signaling rather than in target proteins. However, the pathway analysis of phosphosites should be interpreted cautiously, as this analysis was based on knowledge of biological functions of transcripts and proteins rather than phosphosites themselves. Intriguingly, the vast majority of responsive phosphosites showed equal amplitude changes after LD and HD, indicating that IR doses of 100 mGy are sufficient to trigger maximal phosphorylation (Figure S2F). A subset of phosphosites responded instantaneously after LD and HD radiation, i.e., within seconds (Figure S2D). The observed early responsive phosphosites lacked activated ATM and were not in proteins known to be involved in DNA damage and cell cycle checkpoint signaling, suggesting that instantaneously induced DSBs are unlikely to be the molecular trigger. The temporal dynamics analysis indicated that LD contributed considerably more to early phosphoproteome responses than HD (41% and 13%, respectively). This finding might be compatible with the enhanced induction of ROS after low-dose exposure [15,19,21], illustrated by the LD-specific activation of glycolysis (promoting ROS) and the LD-induced enhanced expression of the redox-sensitive NRF2 pathway, mitochondrial genes and the ROS-regulated major BER AP endonuclease APEX1. Additionally, LD exposure uniquely modulated mitochondrial metabolic proteins and the phosphorylation status of kinases, kinase-substrates and phosphatases predominantly involved in reactive oxygen species (ROS) production [75].

### 4.2. DDR and Mitotic Signaling after LD and HD

A minority of phosphosites (clusters A and B) displayed amplitude differences between LD and HD. Phosphosites in cluster A exhibited increased phosphorylation amplitudes resembling the amplitude changes in activated ATM pS1987 after LD and HD and displayed similar temporal dynamics marked by a time threshold of 5–30 m. This dynamic response might be linked to an increased load of delayed DSBs emerging from radiation-induced labile sites at clustered damage 30–60 min after irradiation [17]. We cannot rule out kinases other than ATM/ATR to phosphorylate cluster A phosphosites. DNA-PKcs appeared to be dispensable, as the dynamics of cluster-A phosphosites are virtually unaltered in irradiated Xrcc5^−/−^ mESCs that lack activated DNA-PKcs [86]. In mammalian cells, the phosphorylation of ATM and ATM-dependent downstream targets (TP53, CHEK1 and CHEK2) is linear over a dose range from 10 mGy to 2 Gy [87,88], respectively. This dose dependency is further substantiated in this study by the dose-dependent phosphorylation of DSB repair factors 53BP1, BRCA1 and MDC1 at multiple sites, closely resembling the dynamics of the autophosphorylation of ATM. The integration of data from this study and the literature suggests dose-dependent and linear activation of the DDR, congruent with the known linear induction of DSB by IR doses [18]. The induction of DDR correlates with a linear dose–response of genetic damage (micronuclei) in the dose range of 10–200 mGy without a threshold, as previously found for human fibroblasts [89] and reticulocytes of irradiated mice [90]. This result suggests that IR induces DNA breaks that are unrepaired and/or irreparable, leading to chromosomal fragmentation in proportion to the dose.

Replication stress induced by DNA inter-strand crosslinks has been shown to result in activation and sustained signaling by ATR, the phosphorylation of FANCI and CHEK1 and a reduction in the firing of dormant origins [91]. HD exposure may also induce replication stress, as we observed robust phosphorylation of FANCI (pS554, pS555 and pT558) and activation of ATR/CHEK1, particularly in DRD cells. Furthermore, HD induced a sharp increase in the phosphorylation of S168 in MDC1, which is essential for its interaction with TOPB1 [92], thereby facilitating ATR activation and CHEK1 phosphorylation at stalled replication forks [26]. Radiation-induced DSB-clustered lesions (approx. 30% after X-rays, [93]) are poorly repaired and thus have an increased probability of encountering replication forks, thereby generating replication stress [94]. Replication stress after HD is in line with the observed proliferation inhibition and enhanced transcription of translesion synthesis polymerases Rev1 and Polk, which are required for restarting replication in damaged DNA. These data suggest either an LD threshold for the induction of replication stress and ATR activation or that the effect of LD is too weak to observe a response.

Proteins in cluster B are enriched for cell cycle terms with preferred targets of mitotic kinases and mitotic phosphatases that control proper cell division [61,62]. Temporal dynamics of cluster B phosphosites mimic those of cluster A (enriched for DDR pathways), albeit in the opposite direction. This is strengthened by similar observations in DRD cells. We uncovered the dose-dependent phosphorylation of cluster A proteins PPP1R7 and ENSA, regulators of mitotic phosphatases PP1 and PP2, respectively. These results suggest that PP1 and PP2 are involved in the activation of the G2/M checkpoint, possibly by the multi-site dephosphorylation of mitotic proteins to diminish ultrasensitivity. Consistently, disrupting phospho-signaling via PPP1R7 (S12 to A12 mutation) dramatically altered the mitotic index and the installment and release of the G2/M checkpoint. Altogether, these data suggest that the dose-dependent transient activation of DDR and the inactivation of mitotic signaling are functionally linked.

### 4.3. P53 and Distinct Gene Expression Programs after LD and HD

Some of the radiation-induced phosphorylations are expected to (de)activate transcription regulators and alter the composition of the produced mRNA species. Nascent transcript (NT) analysis uncovered distinct transcriptional changes following LD and HD radiation. Notably, HD-responsive transcripts were enriched for p53, whereas LD-responsive transcripts were enriched for MYC and POU5F1(OCT3/OCT4)/NANOG transcription regulators. The dose-specificity of nascent transcriptomes might arise from several factors. Firstly, the activation and stability of p53 by phosphorylation are largely dependent on the level of activated ATM, which was substantially higher after HD than LD. Secondly, LD led to enhanced MYC transcription during a 2 h period after irradiation, whereas HD rapidly turned down MYC transcription likely because of high levels of p53 stabilization. The inhibition of MYC and POU5F1/NANOG target gene transcription after HD might be achieved by p53 binding to a distal MYC super-enhancer region [35] and the NANOG promoter [95], respectively. Moreover, within minutes after exposure, HD-mediated phosphorylation rendered MYC/MYCN into a degradable species, and we observed these modifications with a 2 h delay after LD. Increased levels of ROS most likely underlie enhanced MYC transcription and stabilization after LD exposure, as exquisitely ROS-sensitive MAPK kinases [96] drive MYC phosphorylation [19,97].

HD generated a strong p53-dependent transcriptional response (multiple p53 target genes), peaking at 2 h after exposure. There is convincing evidence that p53 expression dynamics can affect cell fate [98,99]; notably, elevated levels of p53 inhibit the transcription of NRF2 and its target genes [100,101] to facilitate apoptosis. These studies support the observation that the NRF2-mediated oxidative stress response is specifically activated by LD-induced ROS and low non-inhibitory levels of p53. A comparable finding was reported for hematopoietic stem cells [15]. Moreover, the concurrency of the inhibition of glycolytic flux with decreased transcriptional activities of MYC and proliferation inhibition is consistent with a recent study showing that MYC controls pluripotent stem cell fate decisions by regulating metabolic flux [102]. After LD, the transcription of MYC and POU5F1/NANOG, which drives cell proliferation and the maintenance of pluripotency, respectively, appeared to continue up to three hours post-IR and may account for the swift recovery of mESCs from the G2/M checkpoint after LD and for the lack of proliferation inhibition. The latter is consistent with the existence of a proliferation inhibition threshold, as reported for LD-irradiated neural stem cells in mice [12].

### 4.4. mTOR/ERK Signaling after LD and HD

In wt cells, the mTOR kinase and substrates were dephosphorylated with similar dynamics (amplitude, kinetics) after LD and HD, possibly to counteract the inhibition of autophagy or the induction of senescence [103,104]. Conversely, ERK1/2 was dose-dependently activated in both LD- and HD-irradiated wt cells. Oxidative stress triggers the ERK1/2-dependent phosphorylation of MYC and regulates MYC stability and degradation [76,77]. The phosphorylation of MYC (pS62) drives MYC-dependent transcription to activate the glutathione-directed survival pathway [105]. ERK1/2 is a chief regulator of Wnt/β-catenin, a pathway known to confer radioresistance to cells [83,106]. ERK1/2 primes phosphorylation of the Wnt inhibitor glycogen-synthase kinase 3 (GSK3-S9), resulting in its inactivation and upregulation of beta-catenin [107]. We observed modest activation of GSK3 (pS9) after LD and HD during 2 h post-irradiation, consistent with the activation of the Wnt/β-catenin pathway. These data suggest that LD differs from HD due to the prolonged transcription of Wnt targets linked to oxidative stress. We hypothesize that the activation of the canonical Wnt pathway contributes to maintaining the self-renewal of mESCs exposed to LD ionizing radiation.

### 4.5. Mitochondrial Function Alterations after LD and HD

Radiation-induced mitochondrial damage results in an altered redox balance and mitochondrial dysfunction [108,109,110,111,112]. In mESCs, LD exposure uniquely up-regulated the transcription of genes involved in antioxidant response, glycolysis, fatty acid metabolism and mitochondrial function (this study), as well as in alterations in mitochondrial proteins and enhanced oxygen consumption rates [75]. These LD-specific responses might arise from mitochondrial dysfunction that promotes perpetual ROS production [15]. Increased ROS production may impair redox balance within cells, triggering the activation of the NRF2 protein [113,114] to promote an antioxidant defense response. We speculate that the absence of mitochondrial dysfunction-related responses after HD may be due to enhanced ATM-p53 signaling after HD along with the saturation of ROS production above 100 mGy [19,21,108], which suppresses antioxidant and mitochondrial responses.

### 4.6. Persistent DSB and Phospho-Signaling

Impairment of DSB repair appears to influence phosphoproteome responses after LD and HD by altering the temporal dynamics or/and amplitude of phospho-signaling. In LD- and HD-irradiated NHEJ-deficient cells, DDR- and mitosis-related phosphosites (clusters A and B) revealed no enhanced amplitude when compared with wt cells, indicating that the initial frequency of DSBs determines the signaling amplitude. However, the impaired repair of DSBs led to prolonged DDR and mitotic phospho-signaling after LD and HD. It is also evident that phospho-signaling after LD extended beyond time points when 53BP1 foci levels returned to control levels and when DSB repair appeared to be completed. In contrast, phosphosites of the mTOR and MAPK/ERK signaling pathways (Clusters C and D) responded in DRD cells with distinctly increased phosphorylation amplitudes and sustained signaling with no temporal and quantitative differences between LD and HD. The fact that it was observed solely in irradiated NHEJ-deficient cell lines suggests a role for low levels of (unrepaired) DSBs. Although the cause for this enhanced signaling remains elusive, it is conceivable that IR-induced ROS [19] might activate the redox-sensitive mTORC1 and ERK1/2/MAPK kinases in DRD cells and drive the phosphorylation of their targets to equal levels after LD and HD.

## 5. Conclusions

In this study, we performed a systems analysis (Figure S1A–J) with a high temporal resolution after exposure to low (0.1 Gy) and high (1 Gy) doses of X-rays in mouse embryonic stem cells (mESCs). In total, we generated over 306 global phosphoproteomes and 30 nascent transcriptomes and assessed dynamic changes in 35,358 phosphosites (in 4994 proteins) and >9000 nascent transcripts at various time points during the 24 h period after DNA damage infliction (Figure S2A–C). Bioinformatics and statistical analyses uncovered 10,181 high-confidence phosphosites (in 2559 proteins) and 2276 genes responsive to IR. To connect these molecular findings with downstream phenotypic changes, we performed kinetic profiling for G2/M checkpoint activation, metabolic flux, and cell proliferation. Our results demonstrate that divergent cellular responses to radiation arise from complex, multi-faceted, dose-(in)dependent, linear and non-linear molecular alterations.

We show that the dose-proportional induction of DNA DSBs, chromosomal damage and the activation of the DDR following IR exposure is congruent with the linear no-threshold model for cancer risk estimation. However, a dose–response relationship was not detected for all molecular responses studied, especially for most IR-induced phosphorylation changes over a post-radiation period of 24 h. Moreover, the integration of gene expression, proteome abundance and metabolic control programs uncovered prominent LD-mediated activation of glycolysis, mitochondrial function and activation of redox-sensitive pathways, suggesting a prominent role for ROS in cellular responses after LD, whereas, after HD, the most outstanding cellular response, i.e., DDR, was triggered by DSB induction. Although increased ROS production may promote tumorigenesis [115,116], it is, however, unclear to what extent these differences in molecular and cellular responses after LD and HD may impact the risk for cancer development notably due to the finding of the dose-proportional (10–200 mGy) induction of cancer-relevant chromosomal damage.

## Data Availability

Mass spectrometry data are available in the ProteomeXchange Consortium with the identifier *PXD013620*. BrU-seq data are available in the GEO repository with the identifier *GSE130066*.

## Supplementary Materials

The following supporting information can be downloaded at: https://www.mdpi.com/article/10.3390/cells11233794/s1, Figure S1: Experimental setup for systems analysis; Figure S2: Phosphoproteome dynamics after LD and HD exposures; Figure S3: DSB repair, DDR signalling and multi-site dephosphorylation signalling in repair-proficient and NHEJ-deficient cells; Figure S4: Proliferation rates of repair-proficient and NHEJ-deficient cells; Figure S5: Phosphoproteome dynamics of NHEJ-deficient cells after LD and HD exposures; Figure S6: Impaired DSB repair rewires crucial signalling pathways; Figure S7: IR dose-dependent nascent transcriptome dynamics; Table S1: Pathway enrichment from phosphoproteins [117,118,119,120,121].
