# Supplementary material for: Divergent Molecular and Cellular Responses to Low and High-Dose Ionizing Radiation
Source: Cells. 2022 Nov 27;11(23):3794. doi: 10.3390/cells11233794 (PMC9739411; doi:10.3390/cells11233794)
Supplement: Supplementary file 1 [file cells-11-03794-s001.zip › Sup figs.pdf]

Fig S1

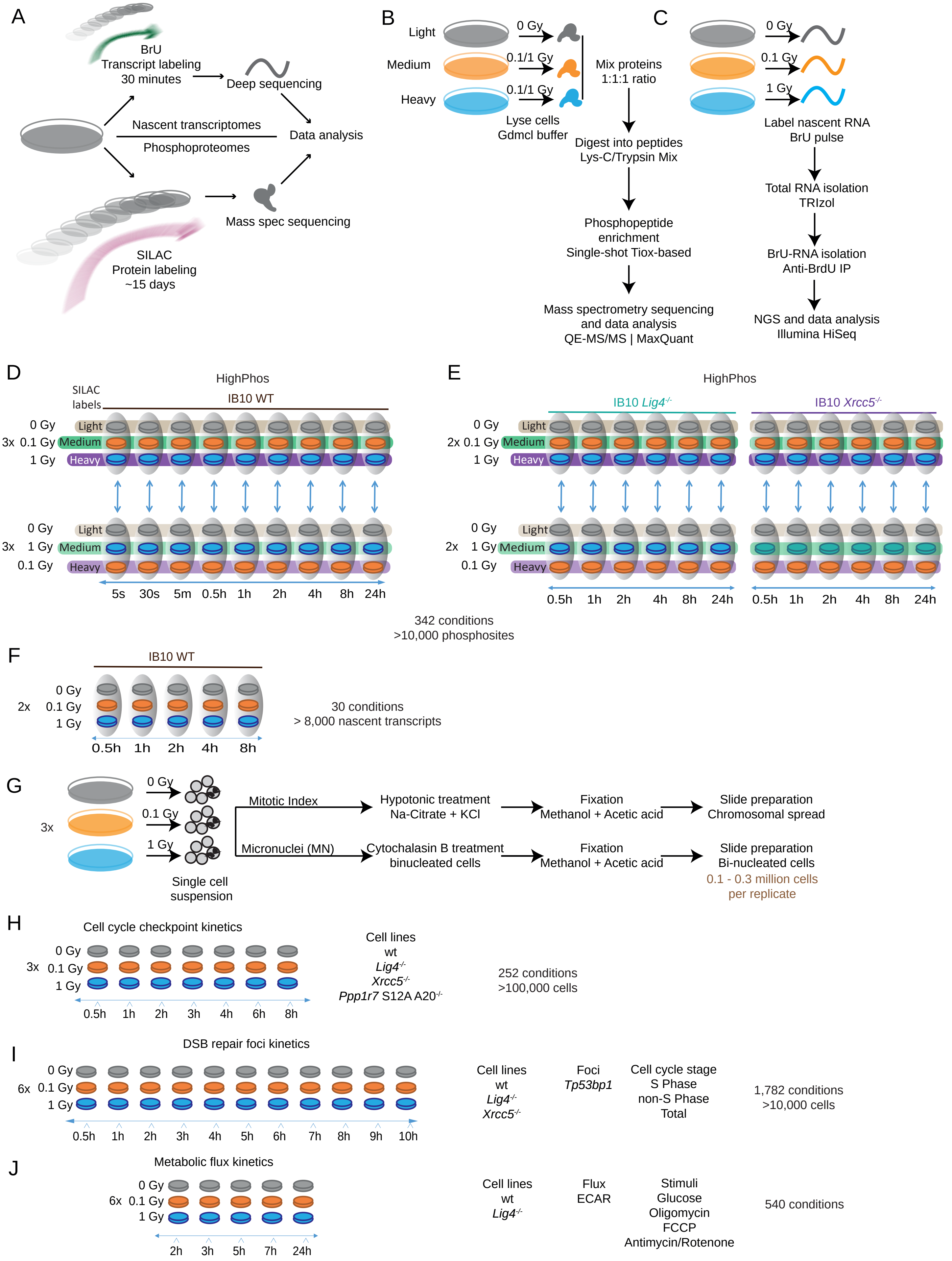
A
BrU
Transcript labeling
30 minutes
Deep sequencing
Nascent transcriptomes
Phosphoproteomes
Data analysis
Mass spec sequencing
SILAC
Protein labeling
~15 days
B
Light
Medium
Heavy
0 Gy
0.1/1 Gy
0.1/1 Gy
Lyse cells
Gdmcl buffer
Mix proteins
1:1:1 ratio
Digest into peptides
Lys-C/Trypsin Mix
Phosphopeptide
enrichment
Single-shot Tiox-based
Mass spectrometry sequencing
and data analysis
QE-MS/MS | MaxQuant
C
0 Gy
0.1 Gy
1 Gy
Label nascent RNA
BrU pulse
Total RNA isolation
TRIzol
BrU-RNA isolation
Anti-BrdU IP
NGS and data analysis
Illumina HiSeq
D
HighPhos
SILAC
labels
IB10 WT
0 Gy
3x 0.1 Gy
1 Gy
Light
Medium
Heavy
0 Gy
3x 1 Gy
0.1 Gy
5s 30s 5m 0.5h 1h 2h 4h 8h 24h
E
HighPhos
IB10 Lig4-/-
IB10 Xrcc5-/-
0 Gy
2x 0.1 Gy
1 Gy
0 Gy
2x 1 Gy
0.1 Gy
0.5h 1h 2h 4h 8h 24h
0.5h 1h 2h 4h 8h 24h
342 conditions
>10,000 phosphosites
F
IB10 WT
0 Gy
2x 0.1 Gy
1 Gy
0.5h 1h 2h 4h 8h
30 conditions
> 8,000 nascent transcripts
G
3x
0 Gy
0.1 Gy
1 Gy
Single cell
suspension
Mitotic Index
Hypotonic treatment
Na-Citrate + KCl
Fixation
Methanol + Acetic acid
Slide preparation
Chromosomal spread
Micronuclei (MN)
Cytochalasin B treatment
binucleated cells
Fixation
Methanol + Acetic acid
Slide preparation
Bi-nucleated cells
0.1 - 0.3 million cells
per replicate
H
Cell cycle checkpoint kinetics
0 Gy
3x 0.1 Gy
1 Gy
0.5h 1h 2h 3h 4h 6h 8h
Cell lines
wt
Lig4-/-
Xrcc5-/-
Ppp1r7 S12A A20-/-
252 conditions
>100,000 cells
I
DSB repair foci kinetics
0 Gy
6x 0.1 Gy
1 Gy
0.5h 1h 2h 3h 4h 5h 6h 7h 8h 9h 10h
Cell lines
wt
Lig4-/-
Xrcc5-/-
Foci
Tp53bp1
Cell cycle stage
S Phase
non-S Phase
Total
1,782 conditions
>10,000 cells
J
Metabolic flux kinetics
0 Gy
6x 0.1 Gy
1 Gy
2h 3h 5h 7h 24h
Cell lines
wt
Lig4-/-
Flux
ECAR
Stimuli
Glucose
Oligomycin
FCCP
Antimycin/Rotenone
540 conditions

Fig S1: Experimental setup for systems analysis

(A) Cells were collected in parallel for both SILAC-based mass spectrometry (MS) and BrU-based RNA sequencing (BrU-seq) experiments. (B) mESCs were SILAC labelled with either "Light", "Medium" or "Heavy" labels and were mock-, LD- or HD- treated respectively and digested into tryptic peptides. Phosphopeptide enrichment was performed using TiO_2 chromatography, sequenced using Q-Exactive MS/MS and analysed by MaxQuant. (C) mESCs were irradiated and incubated for desired time- periods, nascent RNAs were pulse-labelled with 5-Bromo uridine (BrU), followed by immunoprecipitation of BrU-labelled RNA with an anti-BrdU antibody and subsequently sequenced using Illumina HiSeq 2500 sequencers. (D) HighPhos phosphoproteomics experimental set-up of wt cells (left panel) and (E) DRD cells (right panel) resulted in 306 distinct phosphoproteomes. (F) BrU-seq experimental set-up resulted in 30 distinct nascent transcriptomes. (G) High-throughput microscopy (HM) assay workflow for mitotic index and micronuclei experiments. For mitotic index determination, cells were fixed, and chromosomal spreads were prepared to count for mitotic cells. For micronuclei experiments, cells were treated with cytochalasin B to obtain binucleated cells which were subsequently scored for the presence of micronucleus. (H) Overview of the experimental setup for G2/M checkpoint kinetics covering 252 distinct conditions, (I) DNA repair and cell fate foci kinetics covering 3,564 distinct conditions, and (J) metabolic flux kinetics covering 540 conditions. For DNA repair kinetics, cells were fixed with formaldehyde after irradiation and stained for 53BP1 foci with anti-53BP1 antibody; S phase cells were identified with EdU staining. For metabolic flux assays, cells were trypsinized after desired incubation times following irradiation and plated into 96-well XF seahorse plates and incubated in CO_2 free incubator. Real-time extracellular acidification rates (ECARs) were measured using a seahorse XF flux analyser before and after the addition of glucose, oligomycin A, FCCP and Rotenone/Antimycin A in that order.

Fig S2

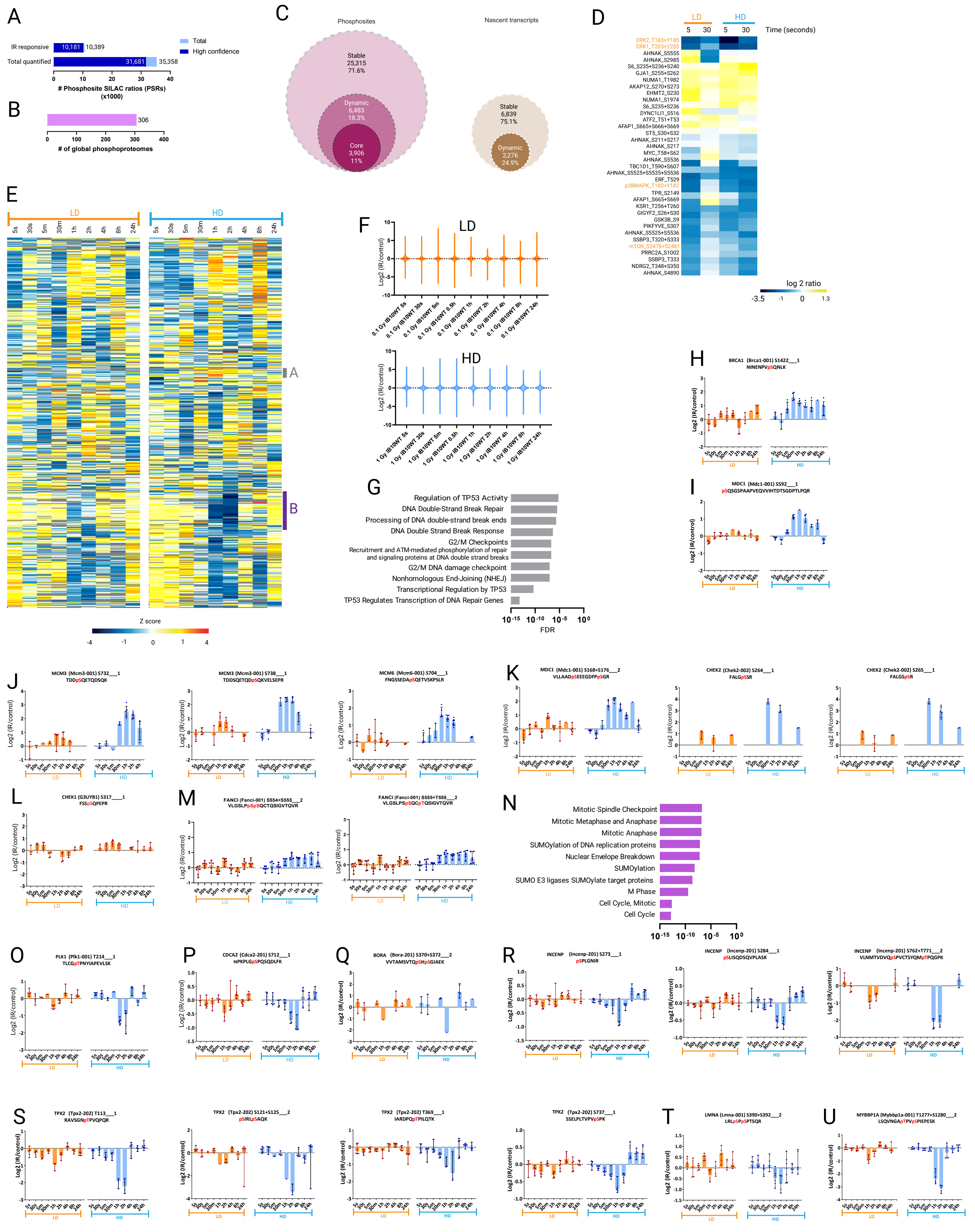

A
IR responsive
Total quantified
Total
High confidence
Phosphosite SILAC ratios (PSRs) (x1000)
B
# of global phosphoproteomes
C
Phosphosites
Nascent transcripts
Stable
Dynamic
Core
D
LD
HD
Time (seconds)
log 2 ratio
E
LD
HD
Z score
F
LD
HD
Log2 (IR/control)
G
Regulation of TP53 Activity
DNA Double-Strand Break Repair
Processing of DNA double-strand break ends
DNA Double Strand Break Response
G2/M Checkpoints
Recruitment and ATM-mediated phosphorylation of repair and signaling proteins at DNA double strand breaks
G2/M DNA damage checkpoint
Nonhomologous End-Joining (NHEJ)
Transcriptional Regulation by TP53
TP53 Regulates Transcription of DNA Repair Genes
FDR
H
I
J
K
L
M
N
Mitotic Spindle Checkpoint
Mitotic Metaphase and Anaphase
Mitotic Anaphase
SUMOylation of DNA replication proteins
Nuclear Envelope Breakdown
SUMOylation
SUMO E3 ligases SUMOylate target proteins
M Phase
Cell Cycle, Mitotic
Cell Cycle
O
P
Q
R
S
T
U

Fig S2: Phosphoproteome dynamics after LD and HD exposures

(A) Statistics displaying the number of phosphosites that were quantified and IR-responsive in all phosphoproteomics experiments. The number in this figure includes all three pairs of SILAC ratio quantifications. (B) The number of biologically distinct phosphoproteomes quantified is represented. (C) Fraction of phosphosite (left panel) and nascent transcripts (right panel) responding to IR. (D) Heatmap of hierarchically clustered log2 SILAC ratios (IR/control) of regulatory phosphosite (PhosphositePlus database) responding instantaneously (5 and 30 seconds) to LD and HD exposures. Values represent a median average of six replicates. (E) Heatmap of hierarchically clustered z-scored log2 SILAC ratios (IR/control) of 9,205 phosphosites that are found to significantly respond to either LD or HD. Phosphoproteomes are ordered from 5s until 24h. The left panel represent LD- and the right panel represents HD-induced phosphoproteomes, respectively. (F) Violin plots displaying Log2 SILAC ratios (IR/control) i.e., amplitudes of phosphosites after LD (upper panel) and HD (lower panel) exposures. Phosphosites are ordered from 5s until 24h. (G) Gene ontology (GO) analysis of phosphoproteins represented in cluster A using Reactome pathways. (H-M) Bar charts showing the kinetics of BRCA1 pS1422, MDC1 pS592, MCM3 pS732 and pS738 and MCM6 pS704, MDC1 pS168+pS176 and CHEK2 pS264 and pS265, CHEK1 pS317 and FANCI pS554+pS555 and pS555+pS558. Dots represent quantifications of up to six replicates. Bars represent median values with a 95% confidence interval (CI) indicated as error bars. (N) GO analysis of phosphoproteins represented in cluster B using Reactome pathways. (O-U) Bar charts displaying the kinetics of PLK1 pT214, CDCA2 pS712, BORA pS370+pS372, INCENP pS273, pS284 and pS762+T771, TPX2 pT113, pS121+pS125, pT369 and pS737, LMNA pS390+pS392, and MYBBP1A pT1277+pS280. Dots represent quantifications of up to six replicates. Bars represent median values with a 95% confidence interval (CI) indicated as error bars.

Fig S3

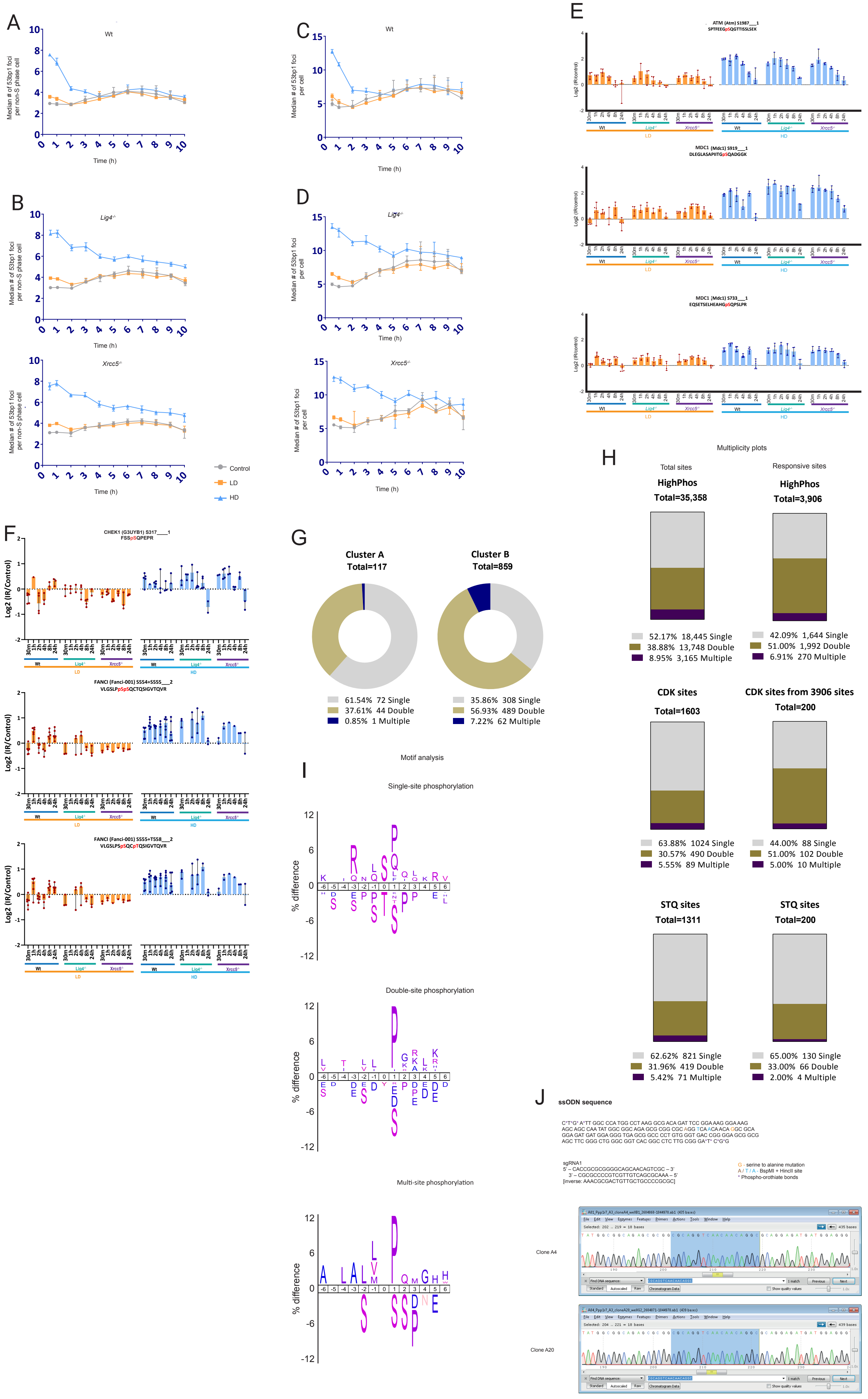

A
Wt
Median # of 53bp1 foci per non-S phase cell
Time (h)
B
Lig4-/-
Xrcc5-/-
Control
LD
HD
C
D
Median # of 53bp1 foci per cell
E
ATM (Atm) S1987___1
SPTFEEGpSQGTTISSLSEK
Log2 (IR/control)
MDC1 (Mdc1) S919___1
DLEGLASAPIITGpSQADGGK
MDC1 (Mdc1) S733___1
EQSETSELHEAHGpSQPSLPR
F
CHEK1 (G3UYB1) S317___1
FSSpSQPEPR
Log2 (IR/Control)
FANCI (Fanci-001) S554+S555___2
VLGSLPpSpSQCTQSIGVTQVR
FANCI (Fanci-001) S555+T558___2
VLGSLPSpSQCpTQSIGVTQVR
G
Cluster A
Total=117
Cluster B
Total=859
61.54% 72 Single
37.61% 44 Double
0.85% 1 Multiple
35.86% 308 Single
56.93% 489 Double
7.22% 62 Multiple
Multiplicity plots
H
Total sites
Responsive sites
HighPhos
Total=35,358
HighPhos
Total=3,906
52.17% 18,445 Single
38.88% 13,748 Double
8.95% 3,165 Multiple
42.09% 1,644 Single
51.00% 1,992 Double
6.91% 270 Multiple
CDK sites
Total=1603
CDK sites from 3906 sites
Total=200
63.88% 1024 Single
30.57% 490 Double
5.55% 89 Multiple
44.00% 88 Single
51.00% 102 Double
5.00% 10 Multiple
STQ sites
Total=1311
STQ sites
Total=200
62.62% 821 Single
31.96% 419 Double
5.42% 71 Multiple
65.00% 130 Single
33.00% 66 Double
2.00% 4 Multiple
Motif analysis
I
Single-site phosphorylation
% difference
Double-site phosphorylation
Multi-site phosphorylation
J
ssODN sequence
C*T*G* A*TT GGC CCA TGG CCT AAG GCG ACA GAT TCC GGA AAG GGA AAG
AGC AGC CAA TAT GGC GGC AGA GCG CGG CGC AGG TCA ACA ACA GGC GCA
GGA GAT GAT GGA GGG TGA GCG GCC CCT GTG GGT GAC CGG GGA GCG GCG
AGC TTC GGG CTG GGC GGT CAC GGC CTC TTG CGG GA*T* C*G*G
sgRNA1
5' – CACCGCGCGGGGCAGCAACAGTCGC – 3'
3' – CGCGCCCCGTCGTTGTCAGCGCAAA – 5'
[inverse: AAACGCGACTGTTGCTGCCCCGCGC]
G - serine to alanine mutation
A / T / A - BspMI + HincII site
* Phospho-orothiate bonds
Clone A4
Clone A20

Fig S3: DSB repair, DDR signalling and multi-site dephosphorylation signalling in repair proficient and NHEJ-deficient cells

Time course mean non-S phase 53BP1 foci in (A) wt, (B) *Lig4*$^{-/-}$ (top panel) and *Xrcc5*$^{-/-}$ (bottom panel) cells and mean total 53BP1 foci in (C) wt, (D) *Lig4*$^{-/-}$ (top panel) and *Xrcc5*$^{-/-}$ (bottom panel) cells that either mock-treated (grey line), LD-exposed (orange-line) or HD-exposed (blue line). Dots represent median values of up to six replicates and error bars represent 95% CI. (E-F) ATM and ATR signalling dynamics in repair-proficient and repair-deficient cells after IR exposure. Bar charts of (E) ATM pS1987 and MDC1 pS919 and pS733 and (F) CHEK1 pS317 and FANCI pS554+pS555 and pS555+pT558 phosphosite kinetics from cluster A in both wt and DRD cells show subtle changes in kinetics. MDC1 pS919 also displayed enhanced amplitude in its phosphorylation in HD-exposed DRD cells compared to wt cells. Dots represent quantifications of up to six replicates. Bars represent median values with a 95% confidence interval (CI) as error bars. (G) Doughnut plots of the multiplicity of phosphosites within cluster A (left panel) and cluster B (right panel). The total number of phosphosites within each cluster is indicated. (H) Bar plots of the multiplicity of the total 35,358 quantified phosphosites (top left) as well as phosphosites with CDK substrate motif (middle left) or (S/T)Q motif (bottom left panels). Similar plots for the IR-responsive total phosphosites (top right), phosphosites with a CDK substrate motif (middle right) or (S/T)Q motif (bottom right panels). (I) Sequence motif analyses and visualisation of 3,906 IR-responsive phosphosites that contained single (top), double (middle) or multiple (bottom) phosphorylation events. Phosphosites that contained single, double or multiple phosphorylation events from the total 35,358 quantified phosphosites were used as background. The phosphorylation amino acid is located at position 0. A p-value cut-off of 0.05 was applied. (J) Optimised, clustered regularly interspaced palindromic repeats (CRISPR)-cas9 genome editing for knock-in (KI) phosphosite mutation. Using CRISPR, precise mutations can be introduced (here SQ->SA) using single-stranded oligodeoxynucleotides (ssODNs) as a template to generate a homology-directed repair (HDR)-dependent KI mutation. However, introducing such KI mutation is quite inefficient and it has been reported that KI rates in mESC are about 2% [117]. Several studies optimised the experimental protocol for improving the efficiency of this method further resulting in 2-40% increase in efficiencies in different cells and animal models [118-121]. Here, we reasoned that using a combination of modified ssODN and co-transfecting it with *Rad51* cDNA could possibly improve the KI efficiency in mESCs. Towards this goal, we designed the ssODNs with a number of modifications. First, we chemically modified the ssODN with eight phosphoorothioates bonds (PS) between the first four and the last four nucleotides to both prevent degradation by endogenous nucleases and being exported out of the cells. Next, we introduced two restriction sites designed so that the two nucleotides in the complementary sequence of both the sgRNA used will be modified. While these restriction sites themselves would help us to measure the KI efficiency by restriction fragment length polymorphism (RFLP) assay, the positioning of these modifications will modify the binding sites of the sgRNAs there by preventing repeated cleavage by Cas9 after KI. Finally, to increase the HDR, we co-transfected a plasmid containing *Rad51* cDNA together with the modified ssODN.

Fig S4

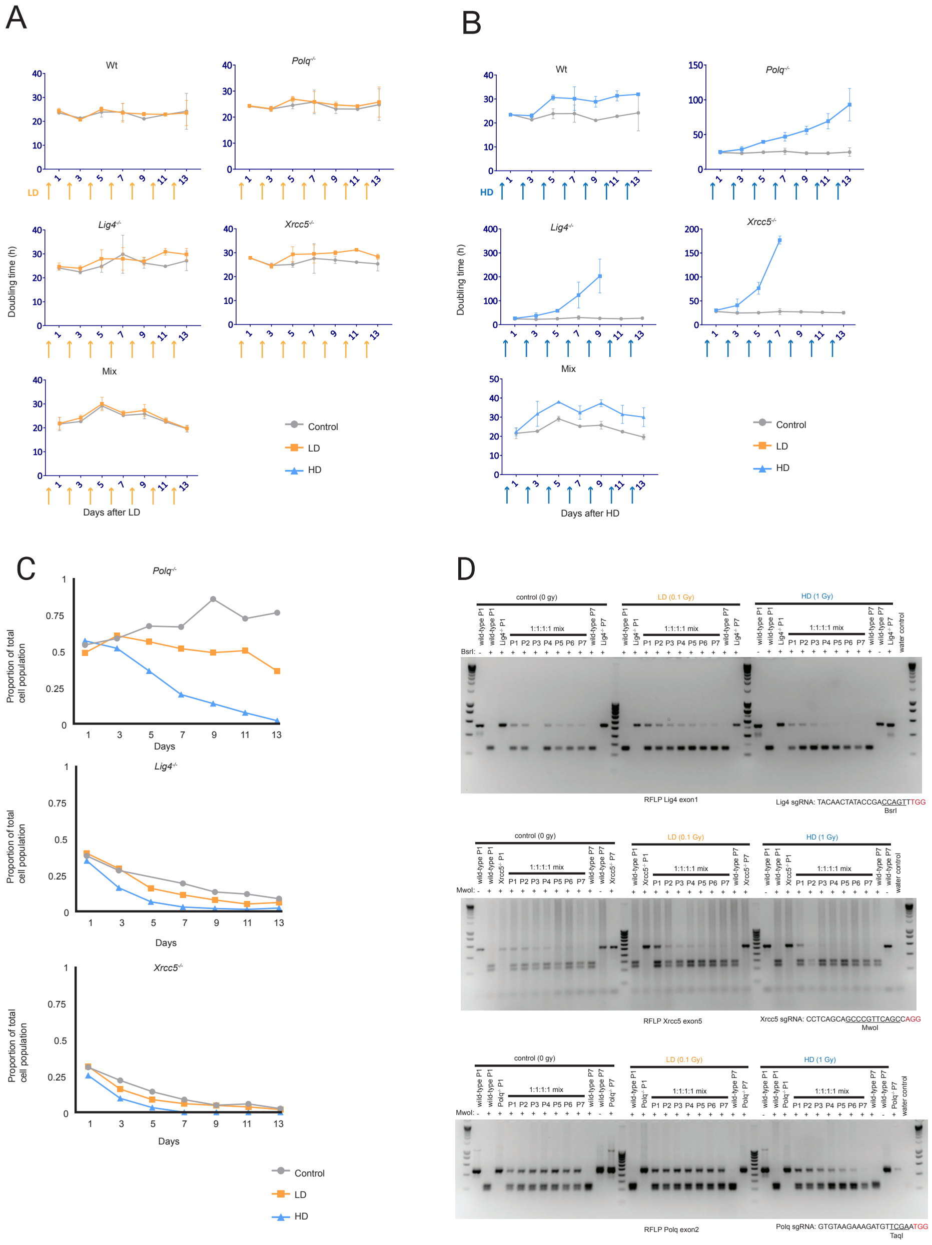
A
Wt
Polq-/-
Lig4-/-
Xrcc5-/-
Mix
Doubling time (h)
LD
Days after LD
Control
LD
HD
B
Wt
Polq-/-
Lig4-/-
Xrcc5-/-
Mix
Doubling time (h)
HD
Days after HD
Control
LD
HD
C
Polq-/-
Lig4-/-
Xrcc5-/-
Proportion of total cell population
Days
Control
LD
HD
D
control (0 gy)
LD (0.1 Gy)
HD (1 Gy)
1:1:1:1 mix
P1 P2 P3 P4 P5 P6 P7
BsrI:
RFLP Lig4 exon1
Lig4 sgRNA: TACAACTATACCGACCAGTTTGG
BsrI
MwoI:
RFLP Xrcc5 exon5
Xrcc5 sgRNA: CCTCAGCAGCCCGTTCAGCCAGG
MwoI
RFLP Polq exon2
Polq sgRNA: GTGTAAGAAAGATGTTCGAATGG
TaqI
water control

Fig S4: Proliferation rates of repair-proficient and NHEJ-deficient cells

(A-B) Proliferation dynamics. Population doubling time of wt, *Polq*$^{-/-}$,*Lig4*$^{-/-}$, *Xrcc5*$^{-/-}$ and mixed population (1:1:1:1) in response to LD (A) and HD (B) over thirteen days. Cells were irradiated every 24h after passaging and trypsinized, counted and reseeded every 24h after irradiation. Grey, orange and blue lines represent unirradiated, LD-exposed and HD-exposed cells, respectively. Dots represent median values of three biological replicates with 95% confident interval (CI) represented as error bars For mixed populations, cell pellets were collected during every passage for RFLP experiments. (C) Detecting subtle changes in cell proliferation rates after LD. Cell pellets from the long-term proliferation of mixed populations containing wt, Polq$^{-/-}$, Lig4$^{-/-}$ and Xrcc5$^{-/-}$ cells (1:1:1:1) were subjected to restriction fragment length polymorphism (RFLP) assay. The loss of a unique restriction site in the knockout clones resulted in a PCR fragment that is resistant to digestion with the corresponding restriction enzyme. The proportion of the loss of these PCR fragments was plotted to obtain competitive proliferation dynamics between the wt and knockout clones in response to LD and HD. (D) Restriction fragment length polymorphism (RFLP) assay. Uncropped gel image showing RFLP experiments. *Lig4*$^{-/-}$ (top panel), *Xrcc5*$^{-/-}$ (middle panel) and *Polq*$^{-/-}$ (bottom panel) cells were either unirradiated or LD- or HD- exposed and DNA samples were digested with corresponding restriction enzymes. The enzymes used are indicated right above the gel image and the sequence of the sgRNAs containing the introduced restriction sites targeted by the enzymes are indicated below the respective gel image. P1 - P7 indicate passage numbers corresponding to day one through day thirteen, respectively.

Fig S5

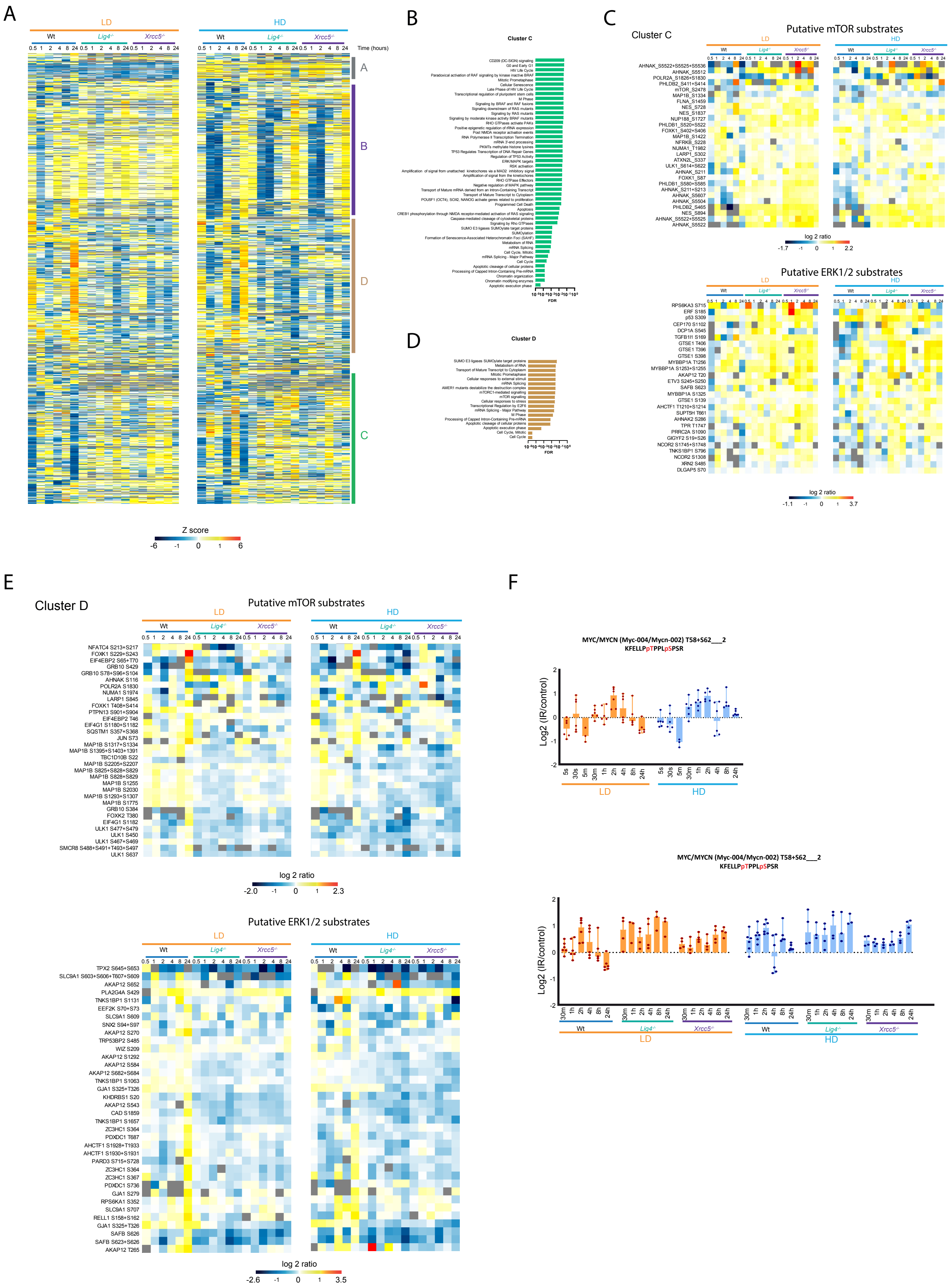
A
LD
HD
Wt
Lig4-/-
Xrcc5-/-
Time (hours)
Z score
B
Cluster C
FDR
C
Cluster C
Putative mTOR substrates
log 2 ratio
Putative ERK1/2 substrates
D
Cluster D
E
Cluster D
Putative mTOR substrates
Putative ERK1/2 substrates
log 2 ratio
F
MYC/MYCN (Myc-004/Mycn-002) T58+S62___2
KFELLPpTPPLpSPSR
Log2 (IR/control)

Fig S5: Phosphoproteome dynamics of NHEJ-deficient cells after LD and HD exposures

(A) Heatmap of hierarchically clustered z-scored log2 phosphosite SILAC ratios (IR/control) of 3,906 phosphosites in repair-proficient and NHEJ-deficient cells that are found to significantly respond either to LD or HD irradiation. Phosphoproteomes are ordered from 0.5h until 24h for wt cells, followed by *Lig4*$^{-/-}$ and *Xrcc5*$^{-/-}$ cells. The left panel represents LD- and the right panel represents HD-induced phosphoproteomes, respectively. (B) Gene ontology (GO) analysis of phosphoproteins represented in cluster C using Reactome pathways with a 0.05 FDR cut-off. (C) Heatmap of hierarchically clustered log2 phosphosite SILAC ratios (IR/control) of putative substrates of mTOR and ERK1/2 kinases in cluster C. Phosphoproteomes are ordered from 0.5h until 24h for wt cells, followed by *Lig4*$^{-/-}$ and *Xrcc5*$^{-/-}$ cells. The left panel represents LD- and the right panel represents HD-induced phosphoproteomes, respectively. (D) Gene ontology (GO) analysis of phosphoproteins represented in cluster D using Reactome pathways with a 0.05 FDR cut-off. (E) Heatmap of hierarchically clustered log2 phosphosite SILAC ratios (IR/control) of putative substrates of mTOR and ERK1/2 kinases in cluster D. Phosphoproteomes are ordered from 0.5h until 24h for wt cells, followed by *Lig4*$^{-/-}$ and *Xrcc5*$^{-/-}$ cells. The left panel represents LD- and the right panel represents HD-induced phosphoproteomes, respectively. (F) Bar charts of MYC/ MYCN pT58+pS62 phosphosite kinetics in both wt and DRD cells show subtle changes in kinetics. Dots represent quantifications of up to six replicates. Bars represent median values with a 95% confident interval (CI) indicated as error bars.

Fig S6

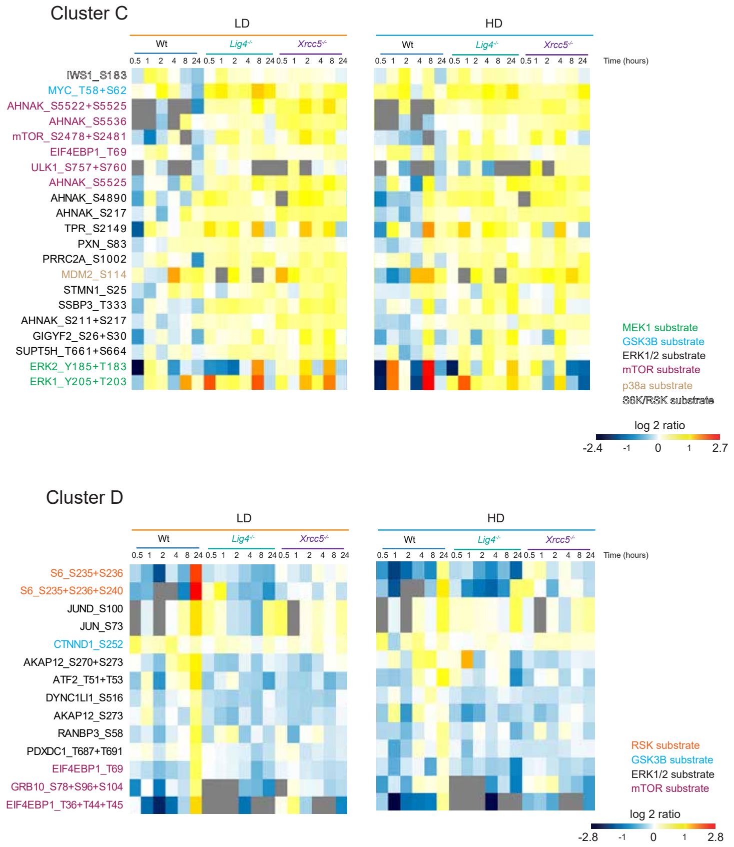

Cluster C
LD
HD
Wt
Lig4-/-
Xrcc5-/-
0.5 1 2 4 8 24
Time (hours)
IWS1_S183
MYC_T58+S62
AHNAK_S5522+S5525
AHNAK_S5536
mTOR_S2478+S2481
EIF4EBP1_T69
ULK1_S757+S760
AHNAK_S5525
AHNAK_S4890
AHNAK_S217
TPR_S2149
PXN_S83
PRRC2A_S1002
MDM2_S114
STMN1_S25
SSBP3_T333
AHNAK_S211+S217
GIGYF2_S26+S30
SUPT5H_T661+S664
ERK2_Y185+T183
ERK1_Y205+T203
MEK1 substrate
GSK3B substrate
ERK1/2 substrate
mTOR substrate
p38a substrate
S6K/RSK substrate
log 2 ratio
-2.4 -1 0 1 2.7
Cluster D
LD
HD
Wt
Lig4-/-
Xrcc5-/-
0.5 1 2 4 8 24
Time (hours)
S6_S235+S236
S6_S235+S236+S240
JUND_S100
JUN_S73
CTNND1_S252
AKAP12_S270+S273
ATF2_T51+T53
DYNC1LI1_S516
AKAP12_S273
RANBP3_S58
PDXDC1_T687+T691
EIF4EBP1_T69
GRB10_S78+S96+S104
EIF4EBP1_T36+T44+T45
RSK substrate
GSK3B substrate
ERK1/2 substrate
mTOR substrate
log 2 ratio
-2.8 -1 0 1 2.8

Fig S6: Impaired DSB repair rewires crucial signalling pathways

Heatmap of hierarchically clustered log2 phosphosite SILAC ratios (IR/control) of phosphosites with known upstream kinases in clusters C (top panel) and D (bottom panel0. Phosphosites texts are coloured according to their upstream kinase.

Fig S7

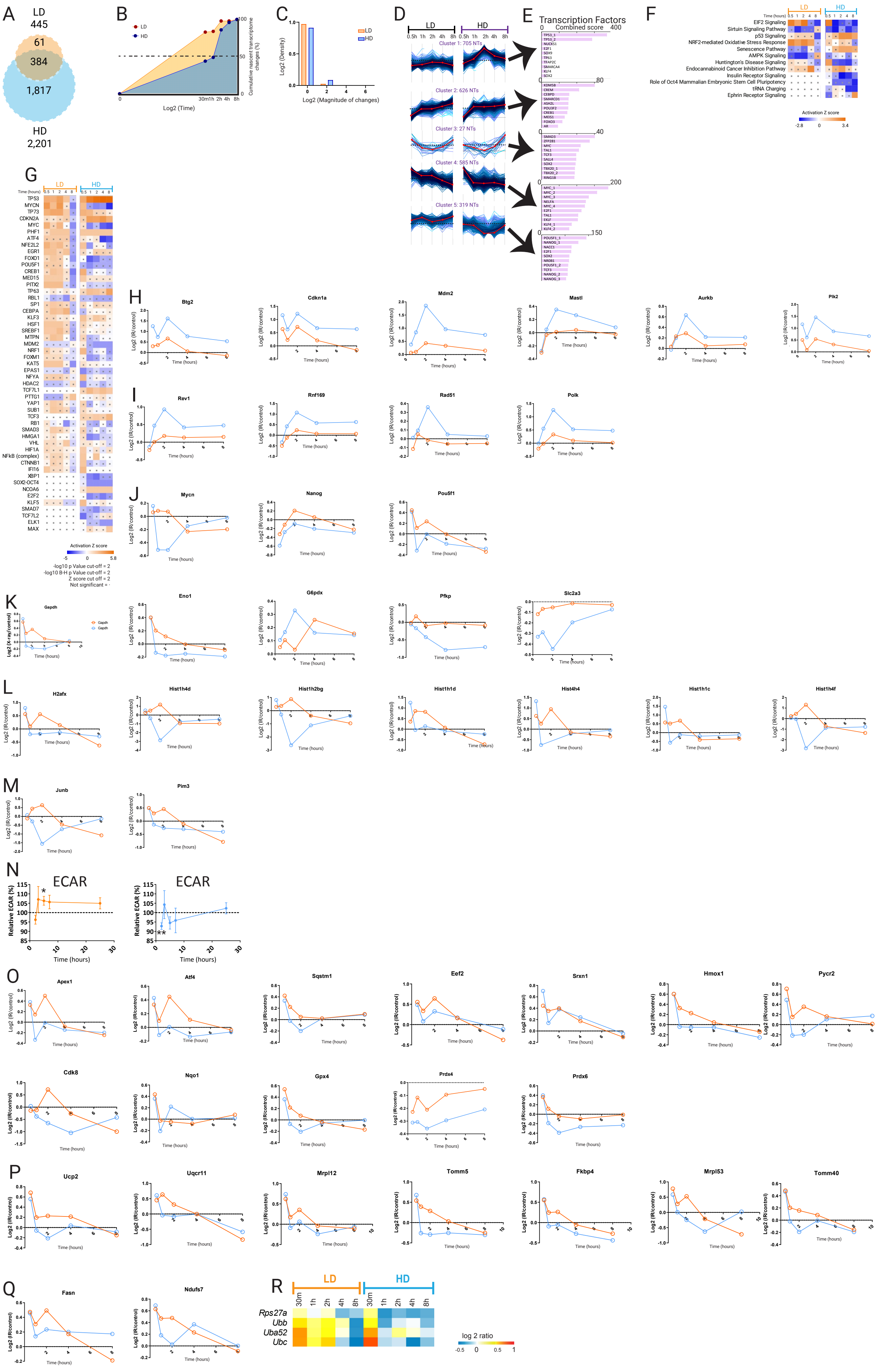

A
LD
445
61
384
1,817
HD
2,201
B
LD
HD
Log2 (Time)
Cumulative nascent transcriptome changes (%)
C
LD
HD
Log2 (Density)
Log2 (Magnitude of changes)
D
LD
HD
Cluster 1: 705 NTs
Cluster 2: 626 NTs
Cluster 3: 27 NTs
Cluster 4: 585 NTs
Cluster 5: 319 NTs
E
Transcription Factors
Combined score
F
EIF2 Signaling
Sirtuin Signaling Pathway
p53 Signaling
NRF2-mediated Oxidative Stress Response
Senescence Pathway
AMPK Signaling
Huntington's Disease Signaling
Endocannabinoid Cancer Inhibition Pathway
Insulin Receptor Signaling
Role of Oct4 Mammalian Embryonic Stem Cell Pluripotency
tRNA Charging
Ephrin Receptor Signaling
Activation Z score
G
Activation Z score
-log10 p Value cut-off = 2
-log10 B-H p Value cut-off = 2
Z score cut off = 2
Not significant = ·
H
Btg2
Cdkn1a
Mdm2
Mastl
Aurkb
Plk2
I
Rev1
Rnf169
Rad51
Polk
J
Mycn
Nanog
Pou5f1
K
Gapdh
Eno1
G6pdx
Pfkp
Slc2a3
L
H2afx
Hist1h4d
Hist1h2bg
Hist1h1d
Hist4h4
Hist1h1c
Hist1h4f
M
Junb
Pim3
N
ECAR
Relative ECAR (%)
Time (hours)
O
Apex1
Atf4
Sqstm1
Eef2
Srxn1
Hmox1
Pycr2
Cdk8
Nqo1
Gpx4
Prdx4
Prdx6
P
Ucp2
Uqcr11
Mrpl12
Tomm5
Fkbp4
Mrpl53
Tomm40
Q
Fasn
Ndufs7
Log2 (IR/control)
Time (hours)
R
LD
HD
Rps27a
Ubb
Uba52
Ubc
log 2 ratio

Fig S7: IR dose-dependent nascent transcriptome dynamics

(A) Venn diagram of nascent transcripts (NTs) that responded to either LD or HD. (B) Temporal dynamics showing the timing of changes, (C) density plot showing the distribution of the magnitude of changes (left panel) of the global nascent transcriptome changes in LD- and HD-exposed cells. (D) Profile plots of five clusters with the median indicated as a red line. (E) Transcription regulators (TRs) were enriched from each of the five clusters. Bars are arranged based on their combined scores. (F) Heatmap of hierarchically clustered activation Z-score (from ingenuity pathway analysis (IPA)) of significantly enriched pathways and (G) upstream transcription regulators for any time point in either LD- or HD-exposed cells. Dots in the blocks represent non-significant values (Z score <2). Line graphs of individual NTs including targets of p53 (H), NTs involved in the DDR genes (I), pluripotency (J), glycolysis (K), histones (L) and proto-oncogenes (M). Orange and blue lines represent the dynamic response to LD and HD respectively. (N) Kinetics of relative extracellular acidification rates (ECARs) after LD (left panel) and HD (right panel). Orange and blue dots represent the median values of six replicates after LD and HD, respectively. Error bars represent the standard error of the mean (SEM). Line graphs of individual NTs involved in the antioxidant response (O), NTs of mitochondrial genes (P) and metabolic genes (Q). Orange and blue lines represent the dynamic response to LD and HD respectively. (R) Heatmap of hierarchically clustered log2 nascent transcript ratios (IR/control) of four ubiquitin-delivering genes showing dynamic response to LD and HD.
